# TMEM123 a key player in immune surveillance of colorectal cancer

**DOI:** 10.3389/fimmu.2023.1194087

**Published:** 2023-06-23

**Authors:** Elisa Pesce, Chiara Cordiglieri, Mauro Bombaci, Serenella Eppenberger-Castori, Stefania Oliveto, Cristina Manara, Mariacristina Crosti, Caner Ercan, Mairene Coto, Andrea Gobbini, Susanna Campagnoli, Tiziano Donnarumma, Manuele Martinelli, Valeria Bevilacqua, Elisa De Camilli, Paola Gruarin, Maria L. Sarnicola, Elisa Cassinotti, Ludovica Baldari, Giuseppe Viale, Stefano Biffo, Sergio Abrignani, Luigi M. Terracciano, Renata Grifantini

**Affiliations:** ^1^ Istituto Nazionale Genetica Molecolare (INGM), Padiglione Romeo ed Enrica Invernizzi, IRCCS Ospedale Maggiore Policlinico, Milan, Italy; ^2^ Institute of Pathology, University Hospital Basel, Basel, Switzerland; ^3^ CheckmAb Srl, Milan, Italy; ^4^ Department of Pathology, European Institute of Oncology, Milan, Italy; ^5^ Department of Surgery, Fondazione IRCCS Cà Granda, Ospedale Maggiore Policlinico, Milan, Italy; ^6^ Department of Oncology and Hemato-oncology, Università degli Studi di Milano, Milan, Italy; ^7^ Department of Biosciences, Università degli Studi di Milano, Milan, Italy; ^8^ Department of Clinical Sciences and Community Health, Università degli Studi di Milano, Milan, Italy; ^9^ IRCCS Humanitas Research Hospital, Rozzano, Italy; ^10^ Department of Biomedical Sciences, Humanitas University, Pieve Emanuele, Italy

**Keywords:** colorectal cancer, tumor microenvironment, tumor-infiltrating lymphocytes, TMEM123, cytoskeleton organization, cell adhesion, migration

## Abstract

Colorectal cancer (CRC) is a leading cause of cancer-associated death. In the tumor site, the interplay between effector immune cells and cancer cells determines the balance between tumor elimination or outgrowth. We discovered that the protein TMEM123 is over-expressed in tumour-infiltrating CD4 and CD8 T lymphocytes and it contributes to their effector phenotype. The presence of infiltrating TMEM123+ CD8+ T cells is associated with better overall and metastasis-free survival. TMEM123 localizes in the protrusions of infiltrating T cells, it contributes to lymphocyte migration and cytoskeleton organization. TMEM123 silencing modulates the underlying signaling pathways dependent on the cytoskeletal regulator WASP and the Arp2/3 actin nucleation complex, which are required for synaptic force exertion. Using tumoroid-lymphocyte co-culture assays, we found that lymphocytes form clusters through TMEM123, anchoring to cancer cells and contributing to their killing. We propose an active role for TMEM123 in the anti-cancer activity of T cells within tumour microenvironment.

## Introduction

The crosstalk between cancer cells and the immune system has gained much attention in recent years (1). Multiple immune-suppressive mechanisms are triggered by the interplay between malignant cells and the surrounding tissue. Cancer cells can actively corrupt their microenvironment by releasing a number of paracrine growth factors, cytokines and metabolites that perturbate signaling and metabolism of surrounding stroma, and mask cancer cells from the immune system to prevent their destruction (2). On the other side neoantigens generated during tumorigenesis could in principle be recognized as foreign and rejected by specific effector T lymphocytes that traffic to the tumor site, where CD8+ T cells mediate direct killing of tumor cells (3). Indeed, tumor-infiltrating lymphocytes (TILs) recruited to the tumor area witness the host’s immune response against cancer cells and have gained increasing attention as prognostic parameters in different cancers (4, 5).

Colorectal cancer (CRC) is a highly heterogeneous disease, with a diverse and plastic immune cell infiltrate. Immune cells present in the tumor-microenvironment (TME) play an important role in modulating tumour growth, progression or elimination. TILs have been associated with lower recurrence and case fatality of CRC, independent of stage. The infiltration of CD8+ T lymphocytes into CRC tumors, has been established globally as a significant predictor of patient prognosis (6, 7). Studies focused on the characterization of TILs in CRC would allow to elucidate the molecular determinants associated with a positive outcome of this cancer.

In this study, we investigated the TMEM123 protein (alias Porimin, KCT-3), a protein that emerged in our study of TILs in solid cancers. We demonstrated that it represents a novel important player in the CRC tumor microenvironment (TME), contributing to cancer immune surveillance. Very little is known about the functional role of TMEM123. TMEM123 (theoretical MW: 21.5 kDa) is a highly glycosylated mucin-like protein with a type 1 plasma membrane topology having 56 predicted glycosylation sites, in particular 47 O-linked (NetOGlyc 4.0), and 9 N-linked (NetNGlyc 1.0). TMEM123 has an extracellular domain with high threonine/serine content, an integral transmembrane domain, and a cytoplasmic tail, endowed by a lysosome/endosome targeting YXXφ motif, which could regulate post-Golgi transport events by forming complexes with adaptor proteins that transport their cargo into transport vesicles (8, 9). TMEM123, was firstly proposed as a pro-oncosis receptor in Jurkat cells (10, 11). In mouse, TMEM123 transcripts were reported to be mainly expressed in mature dendritic cells (DCs), but not in immature DCs, thus TMEM123 was hypothesized to be a new maturation marker for mouse dendritic cells (12).

Overall, we provided new insights on the expression and role of TMEM123 in TILs. We demonstrated that TMEM123 expression in intratumoral-CD8+ T lymphocytes correlated with a better survival for CRC patients. We also showed that expression of TMEM123 is linked to T cell activation and it is required for motility, migration and diapedesis. Our data indicate that TMEM123 is a novel component of T cell protrusions where it plays a key role in clustering of T cell on cancer cells and contributes to T cell effector functions.

## Materials and methods

The Key Resources Table represents a detailed description of the materials and resources used during the experiments described in the manuscript, with the aim of facilitating their reproduction (**Table 1**).

**Table 1:** **Table 1** highlights the reagents, recombinant DNA and oligonucleotides, cell lines, software, and source data essential to reproduce results presented in the manuscript.

REAGENT or RESOURCE	SOURCE	IDENTIFIER
Antibodies		
anti-human IgG- Alexa Fluor 647	Invitrogen Molecular Probes - Thermo Fisher	Cat #A-11013
anti-human-CD62L PE-cy5	BD Biosciences	Cat #555545; DREG-56
anti-Histidin tag	Invitrogen - Thermo Fisher	Cat #MA1-21315; HIS.H8
FAK Antibody Sampler Kit	Cell Signaling	Cat #9330
anti-human IgG-HRP	Invitrogen	Cat #A18805
anti-beta Actin	Invitrogen	Cat #MA1-744; mAbGEa
anti-human-ICAM1 APC	BD Biosciences	Cat #559771; HA58
anti-human-CCR7 PE-Cy7	BD Biosciences	Cat #560922; 3D12
anti-human-CD11b PE	BD Biosciences	Cat #557321; ICRF44
anti-human-CD16 PE	BD Biosciences	Cat #555407; 3G8
anti-human-CD19 PE-Cy5	BD Biosciences	Cat #555414; HIB19
anti-human-CD3 APC	BD Biosciences	Cat #555335; UCHT1
anti-human-CD39 BUV563	BD Biosciences	Cat #748473; TU66
anti-human-CD4 APC-Cy7	BD Biosciences	Cat #557871; RPA-T4
anti-human-CD4-AlexaFluor488	abcam	Cat #ab196372; EPR6855
anti-human-CD44 FITC	BD Biosciences	Cat #555478; G44-26
anti-human-CD56 APC	BD Biosciences	Cat #555518; B159
anti-human-CD69 PE-Cy7	BD Biosciences	Cat #557745; FN50
anti-human-CD8 Pacific Blue	BD Biosciences	Cat #558207; RPA-T8
anti-human-CD8- AlexaFluor488	abcam	Cat #ab196462; EP1150Y
anti-human-E-cadherin	Cell Signaling	Cat #mAb3195; 24E10
anti-human-Ezrin	Cell Signaling	Cat #3145
anti-human-IFNγ PECy5	BD Biosciences	Cat #560742; 4S.B3
anti-human-IL2 FITC	BD Biosciences	Cat #559361; MQ1-17H12
anti-human-IL22 PECy7	BioLegend	Cat #366707; 2G12A41
anti-human-LFA1 APC	BD Biosciences	Cat #559875; HI111
anti-human-PD1 BV711	BD Biosciences	Cat #564017; EH12.1
anti-human-TIM-3 BV650	BD Biosciences	Cat #565565; 7D3
Human Porimin Antibody	R&D systems (Biotechne)	Cat # MAB3010
anti-human-TNFα APC	BD Biosciences	Cat #554514; MAb11
Goat anti-mouse- IgG -AlexaFluor488	Invitrogen Molecular Probes - Thermo Fisher	Cat #A-11001
Goat anti-mouse- IgG -AlexaFluor568	Invitrogen Molecular Probes - Thermo Fisher	Cat #A-11004
Goat anti-mouse- IgG -AlexaFluor647	Invitrogen Molecular Probes - Thermo Fisher	Cat #A-21235
Goat anti-rabbit- IgG -AlexaFluor488	Invitrogen Molecular Probes - Thermo Fisher	Cat #A-11008
Goat anti–rabbit- IgG -AlexaFluor568	Invitrogen Molecular Probes - Thermo Fisher	Cat #A-11011
Goat anti-rabbit- IgG -AlexaFluor647	Invitrogen Molecular Probes - Thermo Fisher	Cat #A-21244
CD3/CD28 Dynabeads	LifeTechnologies-ThermoFisher	Cat #11131D
Goat anti-mouse Star*RED antibody	Abberior Products	Cat # STRED-1001-500UG
Goat anti-rabbit Star*ORANGE antibody	Abberior Products	Cat # STORANGE-1002-500UG
SiteClick™ Qdot™ 705 Antibody Labeling Kit	Invitrogen Molecular Probes - Thermo Fisher	Cat #S10454
SiteClick™ Alexa Fluor™ 647 sDIBO Alkyne	Invitrogen Molecular Probes - Thermo Fisher	Cat #S10911
Actin Nucleation and polymerization antibody sampler kit	Cell Signaling	Cat #8606
Actin reorganization antibody sampler kit	Cell Signaling	Cat #9967
Cofilin activation antibody sampler kit	Cell Signaling	Cat #8354
Paxillin Monoclonal Antibody (GT7612)	Invitrogen Molecular Probes - Thermo Fisher	Cat # MA5-31562
p44/42 MAPK (Erk1/2)	Cell Signaling	Cat #9102
Phospho-p44/42 MAPK (Erk1/2) (Thr202/Tyr204)	Cell Signaling	Cat #9101
Chemical, peptides, and Recombinant Proteins		
Biotin Protein Labeling Kit	Invitrogen	Cat #D20655
Bovine Serum Albumine	Sigma-Merk	Cat #05470
Brefeldin A	Sigma-Merk	Cat #B7651
Cell Trace- CFSE	Invitrogen Molecular Probes - Thermo Fisher	Cat# C34554
Collagenase II	GIBCO-Invitrogen- Thermo Fisher	Cat# 17101015
D-Sorbitol	Sigma-Merk	Cat# S1876
SignalStain^®^ DAB Substrate Kit	Cell signaling	Cat# 8059
DAPI	Invitrogen Molecular Probes - Thermo Fisher	Cat# D1306
1,4-Dithiothreitol	Sigma-Merk	Cat# 3483-12-3
Epidermal Growth Factor	Sigma-Merk	Cat# 11376454001
Ficoll-Paque PLUS	GE Healthcare Life Sciences	Cat# GEHE17-1440-02
Halt^®^ protease inhibitor cocktail	Thermo Scientific	Cat# 78430
H&E Staining Kit (Hematoxylin and Eosin)	abcam	Cat# ab245880
Interferon-gamma	Sigma-Merk	Cat# I17001
Ionomycin	Sigma-Merk	Cat# 56092-82-1
Normal Donkey Serum	Sigma-Merk	Cat# D9663
Normal Goat Serum	GIBCO-Invitrogen	Cat# PCN5000
NucRed	Invitrogen Molecular Probes - Thermo Fisher	Cat# R37106
Percoll solution	GE Healthcare Life Sciences	Cat# GEHE17-0891-02
Phalloidin-AlexaFluor-568	Invitrogen Molecular Probes - Thermo Fisher	Cat# A12380
Phalloidin-AlexaFluor-488	Invitrogen Molecular Probes - Thermo Fisher	Cat# A12379
Primocin	*In vivo*Gen	Cat# ant-pm-05
ProLong Diamond Antifade Reagent	Invitrogen Molecular Probes - Thermo Fisher	Cat# P36961
Phorbol myristate acetate (PMA)	Sigma-Merk	Cat# 79346
Prostaglandine E2	GIBCO-Invitrogen- Thermo Fisher	Cat# P0409
Rapamycin	Sigma-Merk	Cat# 553210
RIPA buffer	Sigma-Merk	Cat# R0278
tumor necrosis factor alpha	Sigma-Merk	Cat# H8916
WheatGermAgglutinine- OregonGreen 488	Invitrogen Molecular Probes - Thermo Fisher	Cat# W6748
Critical Commerical Assays		
ExpiFectamine™ 293 Transfection Kit	ThermoFisher	Cat# A14525
Lipofectamine3000	Invitrogen - Thermo Fisher	Cat# L3000015
Tumor Dissociatiion Kit	Miltenyi Biotec	Cat# 130-095-929
FcR blocking reagent	Miltenyi Biotec	Cat# 130-059-901
Cytofix/Cytoperm	BD Biosciences	Cat# 554714
Rneasy Micro Kit	Quiagen	Cat# 74004
iScript Reverse Transcriptio Supermix	Bio-Rad	Cat# 1708841
SYBR^®^ Green Master mix	Applied Biosystems	Cat# 4309155
Subcellular protein fractionation kit	Thermo Fisher Scientific	Cat# 78840
Pierce™ BCA Protein Assay Kit	Thermo Scientific™	Cat# 23225
Amersham ECL Prime Western Blotting Detection Reagent	GE Healthcare	Cat# RPN2236
Basement membrane extract (BME; Cultrex PC BME RGF type 2)	R&D systems (Biotechne)	Cat# 3533-005-02
Colon carcinoma tissue array (TMA)	Biomax us	Cat# CO1004
PrestoBlue™ Cell Viability Reagent	Invitrogen - Thermo Fisher	Cat# A13261
CellMask™ Green Actin Tracking Stain	Invitrogen - Thermo Fisher	Cat# A57243
LIVE/DEAD™ Viability/Cytotoxicity Assay Kit (Green/Deep Red)	Invitrogen - Thermo Fisher	Cat# L32250
GlycoProfile™II Enzymatic In-Solution N-Deglycosylation Kit	Sigma-Merk	Cat# PP0201
Culture Media and Supplements		
RPMI1640	GIBCO-Invitrogen- Thermo Fisher	Cat# 21875034
RPMI 1640 - without PhanolRed	GIBCO-Invitrogen- Thermo Fisher	Cat# 11835030
Fetal Bovine Serum	GIBCO-Invitrogen- Thermo Fisher	Cat# 26140079
Advanced DMEM/F12	GIBCO-Invitrogen- Thermo Fisher	Cat# 12634028
Expi293™ Expression Medium	GIBCO-Invitrogen- Thermo Fisher	Cat# A1435101
Pennicillin/Streptomycin	GIBCO-Invitrogen- Thermo Fisher	Cat# 15140122
Opti-MEM™ I Reduced Serum Medium	GIBCO-Invitrogen- Thermo Fisher	Cat# 31985070
Experimental Models: Cell Lines		
Expi293	Thermo Fisher	Cat# A14527
HEK293T	American Type Culture Collection (ATCC)	ATCC^®^ CRL-11268™
Jurkat	American Type Culture Collection (ATCC)	ATCC^®^ TIB-152™
HT-29	American Type Culture Collection (ATCC)	ATCC^®^ HTB-38™
Colo205	American Type Culture Collection (ATCC)	ATCC^®^ CCL-222™
HUVEC	American Type Culture Collection (ATCC)	ATCC^®^ CRL-1730™
Recombinat DNA and oligonucleotides		
pcDNA™3.4 TOPO™ TA Cloning Kit	Invitrogen	Cat# A14697
Plasmid encoding full length TMEM123	Genscript	(NM_052932.3) clone ID OHu25599
FANA antisense oligonucleotides (FANA ASOs) and negative controls	AUM BioTech, LLC, Philadelphia, USA	cat # AUMsilence™ sequence: TMEM123 Cat# AUMsilence™: Scramble Control
Hs_GAPDH_1_SG QuantiTect Primer Assay	Qiagen	Cat# 249900; GeneGlobe Id - QT00273322
Hs_PORIMIN_1_SG QuantiTect Primer Assay	Qiagen	Cat# 249900; GeneGlobe Id - QT01029427
Hs_HPRT1_1_SG QuantiTect Primer Assay	Qiagen	Cat# 249900; GeneGlobe Id - QT00059066
Software and Algorithms		
FlowJo	FLOWJO LLC	N/A
GraphPad-Prism	Graphpad	N/A
Fiji (ImageJ)	NIH (https://fiji.sc)	N/A
NIS-Elements v.5.21	Lim-Instruments/Nikon Instruments	N/A
R- Studio	www.r-project.org	N/A

### Cell and culture conditions

Human cell lines were purchased from the American Type Culture Collection (ATCC) and cultured according to standard mammalian tissue culture protocols and sterile technique, in a humidified atmosphere at 37° and 5% CO_2_. Cell culture media were from Gibco-Thermo Fisher Scientific.

### Organoid generation and cultures

Epithelial organoid lines were derived from healthy colon or tumor tissue as previously described (13). In brief, healthy colonic crypts were isolated by digestion of the colonic mucosa in chelation solution (5.6 mM Na2HPO4, 8.0 mM KH2PO4, 96.2 mM NaCl, 1.6 mM KCl, 43.4 mM Sucrose, and 54.9 mM D-Sorbitol, Sigma) supplemented with dithiotreitol (0.5 mM, Sigma) and EDTA (2 mM, in-house), for 30 minutes at 4°C. Colon crypts were subsequently plated in basement membrane extract (BME; Cultrex PC BME RGF type 2, Amsbio) and organoids were grown in human intestinal stem cell medium (HISC), which is composed of Advanced Dulbecco’s modified Eagle medium/F12 supplemented with penicillin/streptomycin, 10 mM HEPES and Glutamax (all Gibco, Thermo Fisher Scientific) with 50% Wnt3a 156 conditioned medium (in-house), 20% R-Spondin1 conditioned medium (in-house), 10% Noggin conditioned medium (in-house), 1x B27, 1,25 mM n-acetyl cysteine, 10 mM nicotinamide, 50 ng/mL human EGF, 10 nM Gastrin, 500 nM A83-01, 3 μM SB202190, 10 nM prostaglandine E2 and 100 μg/mL Primocin (Invitrogen). Tumor specimens were digested to single cells in collagenase II (1 mg/mL, Gibco, Thermo Scientific), supplemented with hyaluronidase (10 μg/mL) and LY27632 (10 μM) for 30 minutes at 37°C while shaking. Single tumor cells were plated in BME and organoids were cultured in HICS minus Wnt conditioned medium and supplemented with 10 μM LY27632 at 37°C.

### Patients and specimens

Tumor tissue samples and matched non-tumoral specimens used (**Table S2**) for *ex vivo* analysis were obtained from the European Istitute of Oncology (Milan, Italy) (the Ethical committee approved the use of specimens for research purposes (permission n. R807/18 IEO 849). FFPE TMA samples used for IHC analysis were derived from archives of Basel Medical Hospital (Ethical approval EKBB 361/12) while FFPE TMA samples used for *in situ* immunofluorescence were from Biomax, inc (Cat N° CO1004).

### TMEM123 cloning, expression and purification

Plasmid encoding full length TMEM123 (NM_052932.3) was purchased from Genscript (clone ID OHu25599). Ectodomain (ECD) of TMEM123 protein was expressed and purified in recombinant form as C-terminal 6-histidine-tagged protein in Expi293T cells. To this aim, TMEM123 ECD was cloned in pcDNA3.4, and the resulting construct was transiently transfected into Expi293 cells (Expi293™ Expression System, ThermoFisher). In brief, 2 µg of construct was used to transfect approximately 2.5× 106 cells/mL in 30 ml culture (95–99% cell viability), using ExpiFectamine293 Reagent, under the manufacturer’s recommendation and cultured for 3 days at 37°C with a humidified atmosphere of 8% CO2 in air on an orbital shaker. Recombinant TMEM123 ECD proteins were affinity-purified by flow gravity of immobilized metal ion affinity chromatography (IMAC). Briefly, supernatant was clarified by centrifugation and was loaded onto a nickel-chelating resin pre-equilibrated with wash buffer (Tris 20mM, NaCL 300mM, imidazole 10 mM pH=8, for of EXPI-TMEM123-ECD and 250 mM imidazole, 50 mM TRIS, 6M urea, 1 mM TCEP, pH 8,5 for *E.coli* TMEM123-ECD). The protein was eluted with the same buffers containing 250 mM imidazole.

### Immunohistochemistry and immunofluorescence analysis of human tissues

Based on TMEM123 immunogenicity results, we investigated TMEM123 expression in cancer and paired non-malignant (NAT) tissues by IHC and immunofluorescence analysis. For IHC, we stained FFPE tissue microarrays (TMAs) available at the Institute of Pathology, University Hospital Basel and the Institute of Clinical Pathology, Basel, Switzerland. All clinic-pathological characteristics are listed in **Table S1** (Ethical approval EKBB 361/12);. HEK293T cells transfected for TMEM123 and mock HEK293T cells were used as positive and negative controls for the optimization of the immunohistochemistry. TMA blocks were cut as 4 μm thick sections. Tissue sections were rehydrated and immunohistochemical staining was performed on a BOND-MAX immunohistochemistry robot (Leica Biosystems) with BOND polymer refine detection solution for DAB, using anti-TMEM123 (clone14A42) antibody. For antigen retrieval, sections were heated in EDTA buffer (pH=9) for 20 minutes at 95°C. TMEM123 (clone14A42) antibody was used at a concentration of 0.24 μg/ml. All sections were counterstained with hematoxylin. The negative control samples were prepared by omitting the primary antibody. Immunoreactivity for TMEM123 in tumor cells was scored semiquantitatively by evaluating the number of positive tumor cells over the total number of tumor cells. Scores were assigned using 10% intervals and ranged from 0% to 100%. Infiltrating lymphocytic and mononuclear stromal cells were counted individually and the results given as absolute number per punch.

For immunofluorescence analysis, we used a commercial colon TMA built from cryo-preserved CRC and normal colon (TMA CO1004, Biomax Inc, containing 40 cases and 10 normal tissue, duplicate cores per case, divided into two identical 50-cores arrays). Sections were fixed in PFA 2% 10 minutes at 4°C, then the aspecific sites were blocked in 10% BSA 1 hour at RT. Tissues were immunostained with Qdot705 (Molecular probes, Thermo Fisher) conjugated anti-TMEM123 mAb, anti-CD8/AlexaFluor488 or anti-CD4/AlexaFuor488 and counterstained with 4’,6’-diamidinio-2- phenylindole (DAPI) plus AlexaFluor-568 conjugated phalloidin (Molecular probes, Thermo Fisher) and mounted with ProLong Diamond Antifade mountant (Molecular probes, Thermo Fisher). Microscopy was performed with an automated Nikon Ti widefield microscope (Nikon Instruments) equipped with a Zyla 4.6 sCMOS camera (Andor) and a 16-led excitation device (PE-4000; CoolLed). 4x, 10x and 20x air objectives (all from Nikon Instruments) were used to acquire images. Large mosaics were composed using the stitching algorithm in NIS-elements AR v.5.2.11.

### TMEM123 silencing or over-expression

TMEM123 was silenced in mammalian cell lines and human T cells by adding to the growth media TMEM123-specific FANA Antisense Oligonucleotides (FANA ASOs) (AUM Biotech) at 10-and 15 µm concentration following the manufacturer’s instructions. Four different FANA ASOs targeting TMEM123 used in this study were designed and synthesized by AUM BioTech, LLC based on the TMEM123 mRNA sequence obtained from NCBI. The following two TMEM123-specific FANA ASOs were selected for the study: TMEM123-3: TGACAATATTCTCACAGTAGC; TMEM123-4:ATACTGCCAACTCTGTTTATC. Scrambled FANA ASO was used as a negative control.

In silico analysis was also performed to obtain the number of potential complementary regions of TMEM123 ASOs in whole human mRNA sequences using GGGenome (http://GGGenome.dbcls.jp/). None of tested sequence have predicted off-target genes. For TMEM123 over-expression, a pcDNA3.1D (Invitrogen) derivative plasmid encoding TMEM123 full-length cDNA was generated and the sequence was verified. HEK293 T cells (400,000/well, in 6-well plates) or the indicated cancer cell lines were transfected with 4 micrograms of the TMEM123 plasmid or with the empty vector as negative control using the Lipofectamine-3000 transfection reagent (Invitrogen) following the manufacturer’s protocol. After 48 h, reduction or increase of TMEM123 expression, was analysed by FACS and RT-PCR analyses. Cell viability was performed with the PrestoBlue cell viability reagent (Invitrogen) according to manufacturer’s instructions.

### Isolation of human CD8+ and CD4+ T cell subsets and TILs

Peripheral blood mononuclear cells (PBMCs) were isolated from buffy coats of healthy donors (in compliance with the Ethical Committee of the Fondazione IRCCS Ca’ Granda Ospedale Maggiore Policlinico, Milan) after density gradient centrifugation with Ficoll-Paque PLUS (GE Healthcare Life Sciences). Cells were then labelled with anti-CD8 Pacific Blue and anti CD4 APC-Cy7 and sorted into CD4+ and CD8+ T cells on a FACS Aria sorter (BD Biosciences). For extraction of TILs, fresh clinical samples from surgical resections were minced with a scalpel and then dissociated into single cell suspensions using the Tumor Dissociation Kit and the gentle MACS Dissociator (Miltenyi Biotec). Afterwards, cells were filtered through 70 μm nylon cell strainers (BD). T cell fractions were recovered after fractionation on a four-step gradient consisting of 100%, 60%, 40%, and 30% Percoll solutions (GE Healthcare).

### Stimulation of T cells

Isolated human T cells were mixed in a 1:1 ratio with Human T-Activator CD3/CD28 Dynabeads (Life Technologies) and cultured for 2 days. After 2 days of stimulation, expression of TMEM123 by silenced or scrambled T cells was determined by FACS analysis and/or qRT-PCR. For cytokine analysis, isolated human T cells were further stimulated with 50 ng/mL Phorbol 12-myristate 13-acetate (PMA) and 1 μg/mL ionomycin (both from Sigma–Aldrich) for 4 hours at 37° in complete RPMI medium. Brefeldin A (Sigma-Aldrich) at 5 μg/mL was added for the last 3 hours. Isolated human T cells were also treated for up to 2 days with: 20 ng/mL IFNγ, 10 ng/mL TNFα, 100 ng/mL Epidermal Growth Factor (EGF), 0,5 M TGFβ, 50 nM Rapamycin.

### T-lymphocyte co-culture with cancer cells or conditioned medium

Cancer cell lines were seeded in 96-well plates (30000/well) and grown to pre-confluency. Human T lymphocytes were added to wells (80000/well) and co-cultured for up to 72h. Alternatively, the conditioned medium of cancer cells was collected from cancer cell cultures and directly added to T cell lymphocyte-containing wells.

### Surface and intracellular staining for flow cytometry analysis

For surface staining, T cells were washed in FACS buffer containing PBS and 5% foetal bovine serum followed by incubation for 20 minutes at 4°C with fluorophore-conjugated antibodies against the several surface markers selected. The following antibodies were used: anti-CD4 APC-Cy7, anti-CD8 Pacific Blue, anti-TMEM123 APC and PE, anti-LFA1 APC, anti-ICAM1 APC, anti-CD44 FITC, anti-CD62L PE-Cy-5, anti-CCR7 PE-Cy-7, anti-PD1 BV711, anti-CD39 BUV563, anti-TIM-3 BV650, anti-CD69 PE-Cy-7 (all from BD Biosciences). Cytokine production was assessed by intracellular staining. The cells were fixed and permeabilized with Cytofix/Cytoperm (BD Bioscience) and then stained with anti-IFN-γ PE/Cy-5, anti-TNFα APC, anti-IL-2 FITC and anti-IL22 PE-Cy-7 (from BD Biosciences). Samples were acquired using a FACS Canto-II flow cytometer (BD Biosciences) and data were analysed using FlowJo software version 10 (LLC).

### RNA extraction and qRT-PCR

Total RNA was isolated using RNeasy Micro kit (Qiagen), reverse-transcribed to cDNA using iScript Reverse Transcription Supermix (Bio-Rad) and amplified using SYBR^®^ Green Master mix (Exiqon). qRT-PCR was performed with QuantStudio (Applied Biosystems), and using the QuantiTect Primer Assay (QIAGEN) for TMEM123. Relative expression was determined using the ΔΔCt method (Livak and Schmittgen 2001), using GAPDH and HPRT as reference for normalization (primers RT2 qPCR Primer Assay for GAPDH and HPRT, QIAGEN). Data were analyzed with the One-Step Plus q-RT-PCR equipment (Applied Biosystems). The experiments were carried out in duplicate for each data point. Normalized data were further referred to an internal experimental control to derive fold change values. Each sample was analysed in duplicates. Results represent at least three replicated experiments.

### Protein extraction and western blot

For extraction of total protein extracts, the indicated cell lines were harvested by scraping into RIPA buffer (Sigma R0278) with Halt^®^ protease inhibitor cocktail (Thermo Fisher Scientific, 78430). After clearing by 10 min centrifugation at 14,000 rpm at 4˚C, the supernatant was collected. For the subcellular fractionation was used the Subcellular protein fractionation kit (Thermo Scientific) following the manufacturer’s protocol. Protein concentrations were determined by the BCA Protein Assay kit (Pierce, 23225). Equal amount of total protein lysates or purified recombinant proteins were separated by pre-cast SDS-PAGE gradient gels (Bolt 4-12% Bis-Tris plus gel, Invitrogen) under reducing conditions, therefore transferred onto nitrocellulose membranes (iBlot Invitrogen). After blocking with 10% dry milk in TBS-T (Pierce, 28358 - 0,1% Tween-20) for 1 hour at RT, the membranes were incubated overnight at 4°C with appropriate dilution of the antibodies in TBS-5% BSA, washed and incubated with HRP-conjugated secondary antibody. Antibody binding was detected using ECL prime Western blotting detection reagents (GE Healthcare) and visualized by iBright imaging system (Thermo Fisher Scientific). ImageJ was used for the densitometric quantification of western blot bands.

### Confocal microscopy of Jurkat cells and T lymphocytes

For confocal microscopy, the indicated cell lines were plated either on Matrigel-coated or on poly-lysine-coated 1.5 thickness coverslips (Corning) and cultured for 24 hours. When needed, cell monolayers were incubated for 10 minutes with live cytoskeleton labelling, either in AlexaFluor-488 or in AlexaFluor-568 (LiveCellMask; Molecular Probes, ThermoFisher Scientific), washed in PBS and then fixed for 15 min in 4% cold paraformaldehyde. After PBS washing, cells were incubated in 10% BSA- 5% Normal Donkey Serum (NDS, Invitrogen) for 1h at RT and subsequently incubated with either Qdot705 or AlexaFluor-647 (both from Molecular probes, ThermoFisher Scientific) conjugated anti-TMEM123 mAb (1:150) for 2 hours at RT. When indicated, cells were also immunostained with anti-LFA-1 antibody or Ezrin antibody (Cell Signaling), followed by secondary antibody labelling conjugated with AlexaFluor dyes (either 488 or 568, all from Molecular probes, ThermoFisher Scientific). Cells were then lightly permeabilized and stained with 4′,6-diamidino-2-phenylindole (DAPI; Molecular probes, ThermoFisher Scientific) to visualise nuclei plus. When detection of cytoskeleton on fixed samples was needed, AlexaFluor-568 or -488 conjugated phalloidin markers (Molecular probes, ThermoFisher Scientific) were used. Stained cells were mounted with ProLong Diamond Antifade mountant (Molecular probes, Thermo Fisher). Fluorescent images were obtained with a confocal laser scanning microscope (Leica SP5 TCS) equipped with 8 laser lines and 4 PMT detectors, using a 63x oil objective (NA 1.43) and LAS-F software v.1.8.5 (all from Leica Microsystem, Germany) or with a video-confocal structure illuminated spinning disk microscope (X-Light-V2/VCS, CrestOptics, mounted on a Nikon Ti microscope, equipped with Andor Du888 EMCCD camera and Andor Zyla sCMOS camera, with 6-laser excitation through LDI laser cube device), using a 100x TIRF oil objective (NA 1.49; from Nikon Instruments Europe). Images were processed with 2D Richardson-Lucy deconvolution to digitally increase confocal resolution, for qualitative representation, using NIS-Elements AR v.5.30 software (Nikon-Lim). For detailed quantifications of morphological parameters and signal intensity and co-localization on raw images, *ad hoc* designed analysis pipelines were employed using NIS built-in general analysis 3 algorithm (GA3). Double-check of correct quantifications for intensity localization were performed using *ad-hoc* developed macros in ImageJ Fiji version (http://fiji.sc) (14).

### Stimulated emission depletion microscopy of CD3 T lymphocytes

Cells were plated on poly-lysine-coated glass coverslips (n. 1.5 thickness; Electron Microscopy) and cultured for 24 hours, then washed and stained. For 1-color STED, cells were stained with anti-TMEM123 antibody followed by hybridization with secondary antibody conjugated with Star*RED (Abberior Products). For two color-STED, cells were also stained with anti-Ezrin antibody followed by hybridization with secondary antibody conjugated with Star*ORANGE (Abberior Products). When needed, also AlexaFluor-488 phalloidin cytoskeleton marker (Molecular Probes, Thermo-Fisher Scientific) was added after a very mild permeabilization step sequential to fixation of secondary antibody stains. Samples were mounted onto glass-slides with ProlongGlass mounting reagent for super-resolution (Molecular Probes, Thermo-Fisher Scientific). Samples were acquired using an Abberior STEDYCON microscope (Abberior Instruments, Germany) for simultaneous confocal and STED microscopy, equipped with a 60x TIRF oil objective (NA 1.49), mounted on an inverted Olympus IX73 microscope, with XY precision stage (Marzhauser, Germany) and Z-stage (PiezoConcept, Germany), with 4 excitation laser lines (405, 488, 561, 640nm) and a 775 nm depletion STED laser (system integrated by Crisel Instruments, Italy). Star*RED and Star*ORANGE fluorophore excitation were kept at 8% and 15% power of the 640nm and 561nm excitation lasers respectively, with both fluorophore depletion obtained at 100% depletion laser power in order to achieve 30nm resolution, at pixel size 15nm, with 7 lines of STED acquisition over the best Z-plan for TMEM123 (parameters were set in order to keep a constant homogeneous photon count of 70photons/frame for both channels in order to allow correct mathematical analysis of photons and channel intensities). A total of n=52 cells were analysed by STED microscopy to observe and correctly localize spatially TMEM123 immuno-labelled molecules relative to Ezrin marker ad to all cytoskeletal protrusions. Acquired images on Abberior Instrument software were saved as.obf files and then opened and analysed *via* ImageJ Fiji version (http://fiji.sc). Raw photon counts were analysed to evaluate signal intensities and co-localization parameters (Pearson Index). Moreover, object segmentation and classification were conducted in order to evaluate and quantify single TMEM_Spots (diameter range 30-50nm) and TMEM_Clusters (range >60nm, <120nm diameter). All localized spots and clusters were further mathematically computed for graphical representation over cellular display, to relatively localize them towards cellular Actin structures and protrusions. An *ad-hoc* created macro of calculations was created on Microsoft excel, employing the localization coordinates of all TMEM spots and TMEM clusters for all analysed cells.

### Live imaging analysis

T cells were isolated from PBMC of healthy donoros, activated with Human T-Activator CD3/CD28 Dynabeads (Life Technologies), and cultured for 2 days. Afterwards, cells were sorted for CD8+/TMEM123+ or CD4+/TMEM123+, labelled with CFSE, then incubated in the optical imaging 96-well plate pre-seeded with cancer cells. Cell interactions were followed in timelapse over 65 hours with 3 hours loop (time frame 3h, duration 65h) employing a fully-automatized customized spinning disk confocal microscope equipped with a X-Light V2 spinning head (CREST Optics, Rome, Italy) mounted on a Nikon Ti inverted microscope (Nikon Instruments), with an Andor DU888 EM-CCD camera (Andor) for high efficiency/high resolution detection and a SpectraAura 6-Led excitation device (Lumencore), using a 20x objective (Nikon Instruments, NA 0.95) enabled for perfect focus maintenance over long-lasting live experiments. Temperature (37°C) and CO2 concentration (5% in air) were maintained thanks to the incubator cage of the system (OKO-Lab, Pozzuoli, Italy) and checked by software (specialized OKO-Lab driver installation in Nikon NIS-Elements AR v5.2.11 software). At the end of the live acquisition, the cells were fixed, stained with surface markers antibodies (anti-CD3, anti-CD4, anti-CD8 and anti-TMEM123 mAbs) and then acquired at high confocal resolution and super-resolution, employing a VCS structure illumination module (lateral resolution 110nm, z-resolution 270nm; CREST Optics), a TIRF 100x objective (NA 1.49, Nikon Instruments) and an Andor Zyla sCMOS camera (Andor). Motility analysis was conducted using NIS-Elements AR v.5.2.11 software, employing the Cell Motility and the General Analysis 3 modules. In brief, signal positive cells (for CFSE signal only, for TMEM signal only, for both signals) were binarized and segmented as objects over time. In order to better delineate the perimeter of each cell using growing algorithms, also DIC images were exploited to derived polarized membrane structures. Each object, classified for labelling and growing-algorithm-derived morphology, was quantified for its morphology over time and primary morphological parameters, such as centroid and equalized diameter, were automatically used to detect and calculate object movement trajectories over space and time. Secondary parameters, such as velocity, speed, acceleration and path length were used to develop graphic interfaces to visualize cell movement in space (scatter trajectory plot) and in time (histogram and line plots). For CD4+ TMEM123+ cells, over 9 analyzed FOVs with n=344 cell trajectories were quantified and plotted; for CD8+ TMEM123+ cells, over 9 analyzed FOVs with n=785 cell trajectories were quantified and plotted.

### Boyden chamber migration and endothelial transmigration assays

For transwell migration assay, 10^5^ CFSE-labelled Jurkat cells or CD8 T cells (silenced or scrambled for TMEM123) were seeded in serum-free media in the upper chamber of transwell inserts with 3.0 μm pore size membrane (24-well format, Costar, Corning Incorporated). The lower chambers were seeded with either HT-29 or Colo205 cells or simply filled with RPMI-10% FBS. Live imaging was conducted using a customized spinning disk microscope with a X-LightV2 spinning Head (CREST Optics) mounted over an inverted Ti microscope (Nikon Instruments), using 6-LED excitation (SpectraAura; Lumencore), an EM-CCD camera (DU888; Andor) and a 4x objective (Nikon Instruments), in an incubator cage (OKO-lab) with centralized monitoring of temperature and CO_2_ (via NIS-Elements AR v.5.2.11). Live acquisition was conducted over whole well (employing large image 5x5 FOVs mosaic per well) in Z-stack mode to acquire both the upper chamber and the bottom chamber in each well, over a 15-minute loop of time frame, for a total duration of 18 hours. Cells migrating towards the lower chambers were quantified *via ad-hoc* designed analysis pipeline using the NIS-Elements AR module General Analysis 2. In brief, cellular objects defined by CFSE signal intensity, visualized in upper and bottom chamber at each time point, were binarized and segmented to evaluate correct quantification. Objects better visualized at the level of best focal plan in bottom chamber were used to define the effectively transmigrated cells. The trans-endothelial migration assay was set-up and acquired similarly. In details, 10^5^ HUVEC cells were seeded in the upper chamber of transwell inserts with 3.0μm pore size membrane (24-well format, Costar, Corning Incorporated), and grown till confluency. 7.5x10^4^ Jurkat cells or CD8 T cells (silenced or scrambled for TMEM123) were labelled with Nuc Red (Life Technologies) and then seeded on top of the CFSE-labelled HUVEC monolayer in RPMI medium without phenol red and FBS. The lower chamber of the inserts was filled with HT-29 conditioned medium. Cells reaching the lower side of the membrane, acquired as best focal plan within each well Z-stack, were quantified using the above-described analysis pipeline.

### CRC organoids T cell co-cultures and live imaging

Organoids were split and digested a 5 to 7 days prior to co-culture and seeded at a density of 5000 cells per 10 μL of BME (25,000 cells per well in) in a 96-well optical culture plate. Two days prior to co-culture, CD8 T cells were sorted from CRC matched patient PBMCs and *in vitro* activated with anti CD3/CD28 beads for 48h in presence of silencing TMEM123 FANA-aso or scramble FANA unrelevant control. Activated CD8 T cells were then labelled with Qdot705-conjugated anti-TMEM123 antibody and CFSE and added to cultured wells of organoids at the density of 50000 cells/well. Alternatively, T cells were labelled with live-cell nuclear Hoechst labelling (Molecular Probes, Thermo-Fisher Scientific) and CRC organoid were labelled with Green-Live/DeepRed-Dead viability dye (Molecular Probes, Thermo-Fisher Scientific). Experiment was performed on duplicate culture samples for each treatment condition (TMEM123 FANA or scramble FANA) plus duplicate conditions on only Matrigel drop not containing CRC organoids, to evaluate matrigel/media-induced T cell migration. Moreover, several wells with cultured CRC organoids were also monitored over time without co-culturing of T cells, to evaluate vitality of CRC cells and organoids in the imaging set-up condition. Co-cultures were monitored in timelapse over 7 days with 1 hour loops (168 total loops) employing a fully-automatized customized spinning disk confocal microscope equipped with a X-Light V2 spinning head (CREST Optics, Rome, Italy) mounted on a Niknn Ti inverted microscope (Nikon Instruments), with an Andor DU888 EM-CCD camera (Andor) for high efficiency/high resolution detection and a SpectraAura 6-Led excitation device (Lumencore), using both a 4x and a 20x objective (Nikon Instruments), both enabled for perfect focus maintenance, at all time-points, thanks to the *ad-hoc* customized JOBS (Lim-instruments) computerized pipeline of acquisition. At each time frame, all wells were acquired in 4-frame mosaic mode at 4x, in thick Z-stack volume (800 µm) to allow total cell count per well area and volume, and in two specific areas for each well at 20x (over a 300 µm Z-stack), to highlight the two chosen best organoids in each cultured well and follow the directed migration of T cells. Temperature (37°C) and CO2 concentration (5% in air) were maintained thanks to the incubator cage of the system (OKO-Lab, Pozzuoli, Italy) and checked by software (specialized OKO-Lab driver installation in Nikon NIS-Elements AR v5.2.11 software). All CFSE+ CD8+ T cells and TMEM123+CFSE+ CD8+ T cells migrated through the Matrigel drop in each well, were counted *via ad-hoc* designed analysis pipeline using the NIS-Elements AR module General Analysis 2. In brief, binary areas were designed to determine the total well area, the Matrigel drop area, and the border area at the marginal zone of the Matrigel drop. Cellular objects, defined by CFSE and TMEM123 intensity levels *via* thresholding algorithms and morphological parameters, were tracked and quantified over time to evaluate the correct number and ratio of migrating T cells within organoid milieu. Organoid structures (segmented *via* EDF processing and DIC-homogenation algorithm) were also quantified over time for size variation and their cancer cells were segmented by size and light filtering to be quantified in time. Specific tracking algorithms for T cell motility, similar to those above explained, were also applied to follow the migration of TMEM123+ CD8+ T cells towards the organoid in 20x-magnified time-lapse imaging. Primary morphological parameters (centroid, equalized diameter) were used for cell tracking and displacement over time to develop direction migratory plot and binarized trajectories (Cell motility module, NIS-Elements AR v5.2.11). At the end of the live acquisition, the co-cultures were fixed, pending whole-mount staining procedure.

### High resolution whole-mount imaging of CRC organoids-T cell co-cultures

Fixed co-cultures of CRC tumoroids were stained in whole-mount to evaluate the cellular interactions among TMEM+ CD8 T cells and cancer organoids. In parallel also control CRC tumoroids were stained for epithelial marker (pan-keratin) and cellular proliferation (Ki-67). In brief selected wells of the live-monitored co-cultures were permeabilized in 0.5% Triron X-100 in PBS for 3h, prior to overnight aspecific immune-site blocking in 10% BSA + 5% FCS in 0.2% Triton X-100 in PBS. Samples were then immune-stained with antibodies against Pan-keratin (ab9377, Abcam, 1): followed by its secondary antibody labelling conjugated with AlexaFluor-568 (MolecularProbes, Thermo Fisher Scientific) and directly-fluorescent labelled antibodies for TMEM123 (Qdot-705) and CD8 (AlexaFluor-488) and counter labelled for nuclear staining with DAPI (Thermo Fisher Scientific). Every whole-mount staining passage was conducted over long-lasting steps to allow correct labelling penetration in thick specimen. Finally, samples were acquired at high and super-resolution, employing both a Leica SP5 laser scanning confocal microscope (Leica Microsystems, Germany), using a 10x air objective, and a multi-purpose Nikon Ti microscope equipped with spinning disk confocal head and a VCS structure illumination module (X-Light-V2+VCS; CREST Optics, Rome, Italy), using low magnification objective with high NA to obtain crisp, resolved images through thick specimen. Z-scanning was performed at ranges of 0.5 - 2.5 µm step over 300 µm volumes. VCS algorithm M3 was used for super-resolution deconvolution (CREST Optics), followed by Richardson-Lucy 3D deconvolution (NIS-Elements v5.2.11) for better qualitative image 3D visualization.

### Statistical analysis

In IHC analysis, the expression levels of TMEM123 in the several tissues and cell types were compared by means of the Wilcoxon Mann Whitney U test and correlation coefficients calculated according to Spearman. Survival curves were depicted according to the Kaplan-Meier method and compared with the log rank test. Analyses were conducted on GraphPad Prism (V7) and R (Version 3.4.1 (2017–06–30), or higher www.r-project.org). For all other tests, difference among groups were analyzed using the two-tailed X2 test, the Student’s t test, and ANOVA using GraphPad software.

## Results

### TMEM123 is detected both in cancer cells and in tumor-infiltrating immune cells

**Figure 1:**
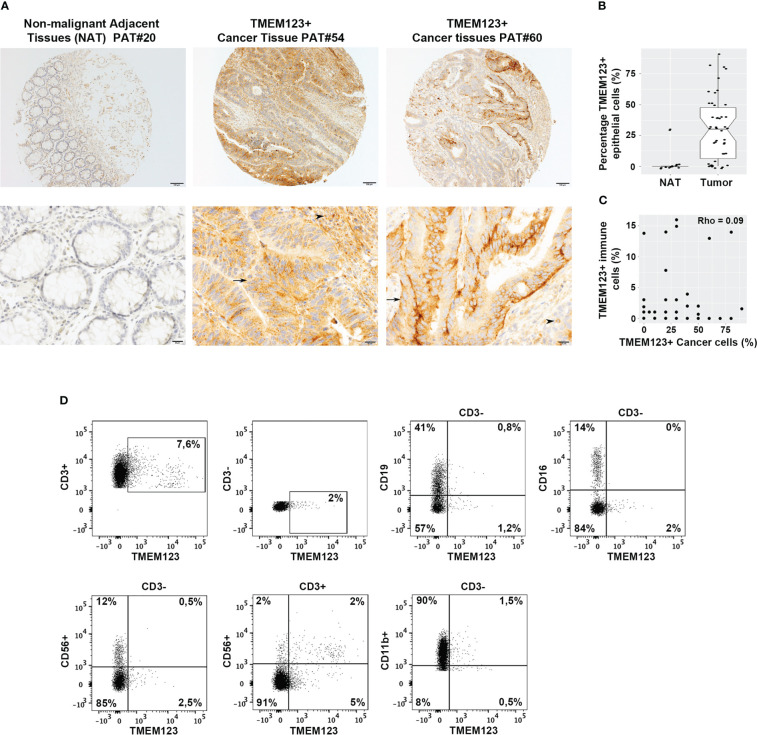
TMEM123 is over-expressed in CRC tissues and in tumor-infiltrating immune cells. **(A)** CRC TMA1 (see **Table S1**) was stained with an anti-TMEM123 monoclonal antibody. Black arrows indicate positive tumor areas and black arrowheads point to positive immune cells, which include both lymphocytes and macrophages, surrounding the tumor. **(B)** Boxplots of detected IHC scoring values in NAT and malignant tissues showed that TMEM123 is expressed almost exclusively in cancer tissues compared to the paired NAT (P<0.0001). **(C)** Scatter plot representation of IHC data showing negative correlation between TMEM123 positivity in cancer cells vs in infiltrating immune cells. **(D)** Flow cytometry analysis of major tumor-infiltrating immune cell populations. TMEM123 cell surface expression was assessed by FACS in CD3+T lymphocytes, CD19+ B lymphocytes, CD3-CD16+ and CD3-CD11b myeloid cells, CD3-CD56+ NK cells, CD3+CD56+ NKT cells isolate from tumor-infiltrating immune cells. Dot plots show representative staining of one clinical sample of three analyzed with similar cell distribution.

We first investigated TMEM123 expression in CRC by immunohistochemistry (IHC) on formalin-fixed paraffin-embedded (FFPE) tissue microarrays (TMAs) representing CRC and paired non-malignant adjacent tissues (NAT) (referred as TMA1, **Table S1**), using TMEM123-transfected and mock HEK293T cells as positive and negative staining controls (**Figure S1A**). IHC was conducted using an *ad hoc* generated monoclonal antibody (mAb) raised against recombinant His-tagged TMEM123 ectodomain (ECD) produced in Expi293 human cells (**Figures S1A-D**). In all tissues, we observed an intense staining of TMEM123 on the membrane and partially in cytosols of the malignant cells, which was absent in almost all paired NAT (**Figures 1A, B**). Furthermore, we observed TMEM123 positive staining in immune cells surrounding the tumor (**Figures 1A, B**). The TMEM123 positive immune cells were haphazardly distributed throughout the tissue samples, as in some samples they appeared to be very close to the neoplastic cells but in other samples they were either clustered around very tiny blood vessels or scattered in the stroma. Interestingly, we found that TMEM123 positivity in cancer cells did not correlate with positivity of infiltrating immune cells (Rho=0.09; **Figure 1C**). Moreover, we investigated whether TMEM123 expression was evenly distributed among tumor-infiltrating immune cells or was enriched in specific ones. Immune cells were isolated from CRC samples (N=3) and TMEM123 expression was assessed by FACS analysis on the major subset populations, based on their immunologic function and cellular phenotyping expression: T, B and Natural Killer (NK) lymphocytes and myeloid cells. The CD3+ T lymphocyte population showed the highest frequency of TMEM123 positive cells (approximately 8%). A fraction of TMEM123 positive cells was also found in the CD3+ CD56+ population, while all other tested cell types displayed a low positivity rate (≤2% of cells) (**Figure 1D**). As far as the CD3+ CD56+ population is concerned further investigations are needed to understand whether these are NKT or other lymphocyte populations expressing CD56.

### TMEM123 is expressed in TILs, with a highest frequency in CD8+ T cells and is associated to better survival rate

**Figure 2:**
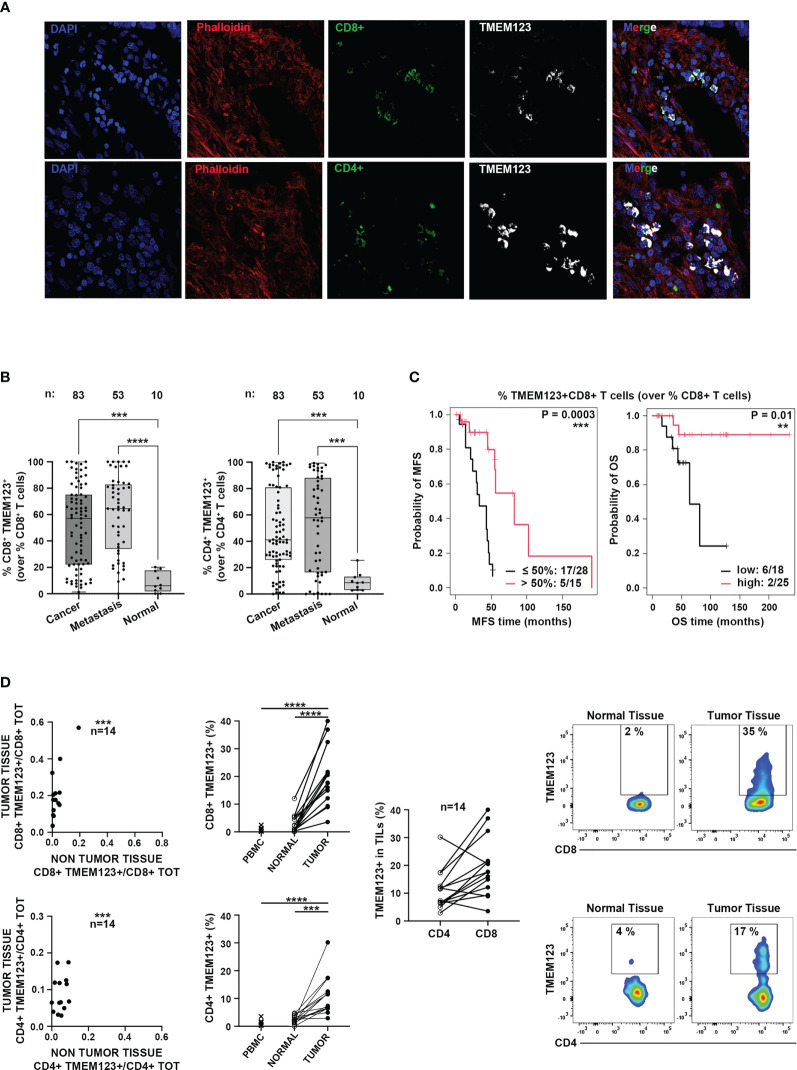
TMEM123 expression in intratumoral-CD8+ T lymphocytes correlated with better patient survival. **(A)** Representative qualitative immunofluorescence analysis of cryopreserved CRC tissues. TMEM123 expression is detected in intratumoral CD8+ (green, left) and CD4+ (green, right) T lymphocytes. **(B)** Boxplots represent the quantification of merged fluorescence signals of TMEM123 positive CD4+ (left) and CD8+ (right) T lymphocytes, assessed in FFPE tissue samples from two different tissue micro-arrays (see **Table S1**) containing CRC at different stages and normal tissue cores (n:83 primary cancer; n:53 metastases; n:10 normal tissues). **(C)** Kaplan–Meier analysis of metastasis-free survival (MFS) and overall survival (OS) stratified according to the presence (red line) or absence (black line) of TMEM123 in tumor-infiltrating CD8+ T lymphocytes. **(D)** FACS analysis of TMEM123 expression in TILs. Left graphs show the correlation between TMEM123+ lymphocytes present in cancer vs normal tissues and the TMEM123 positivity (%) in CD4+ and CD8+ T cells detected in tumor, non-tumor tissues and PBMC of each patient. The central graph shows the percentage of tumor-infiltrating TMEM123+CD4+ and TMEM123+CD8+ T cells detected in the same patient. Right graphs: representative FACS staining for TMEM123 expression in tumor-infiltrating CD4+ and CD8+ T cells compared to normal tissues. Statistical significance is denoted by asterisks (**= <0.01; ***=<0.001; ****= <0.0001).

Due to the relevance of tumor-infiltrating T lymphocytes in the anti-cancer immune response, we further assessed TMEM123 expression in CD8+ and CD4+ T lymphocytes infiltrating CRC tissues by multiple immunofluorescence confocal microscopy on cryopreserved CRC tissues (N=4 samples). A qualitative analysis of fluorescence signals revealed high TMEM123+ expression on CRC-infiltrating CD4+ and CD8+ T lymphocytes (**Figure 2A**). To better appraise the frequency of TMEM123 positive lymphocytes in relation to cancer progression, we carried out a multiple immunofluorescence staining on a FFPE tissue microarray containing other two sets of clinical samples. We stained a commercial FFPE CRC TMA carrying 40 primary cancerous and 10 normal tissues (TMA Biomax in **Table S1**) and another FFPE CRC TMA (CRC TMA2 in **Table S1**) representing tissues of different colon cancer stages (43 primary cancer with paired synchronous metastases or local recurrences or metachronous metastases) for which follow up and survival data were available. Quantification of merged fluorescence signals for TMEM123, CD4 and CD8, in the 83 tested primary tumors confirmed a higher TMEM123+ expression in CRC-infiltrating CD4+ and CD8+ T lymphocytes compared to normal colon lymphocytes, while the frequency of TMEM123+ TILs in cancer and corresponding metastases was similar (**Figures 2B**, **S2**). Remarkably, TMEM123 expression in intratumoral-CD8+ T lymphocytes correlated with better survival rate for the patients, in terms of both metastases free and overall survival (MFS, P=0.0003, OS P=0.01) (**Figure 2C**).

Finally, we assessed expression of TMEM123 by FACS analysis in CD4+ and CD8+ T lymphocytes isolated *ex vivo* from paired cancerous and non-tumoral tissue samples (proximal, but not adjacent to cancer cells) (N=14) and from peripheral blood (N=11) (**Table S2**). TMEM123 expression was found in both tumor-infiltrating CD8+ (19% ± 3%) and CD4+ (11% ± 2%) T cells, while it was almost undetectable in T lymphocytes isolated from normal tissues (2.7% ± 0.5%) or PBMCs (0.9% ± 0.2%). In the majority of patients tested by FACS (78%) TMEM123 had a higher expression in tumor-infiltrating CD8+ than in CD4+ (**Figure 2D**). Collectively, these observations supported the hypothesis of a functional role of the protein in tumor-infiltrating T cells.

### TMEM123 expression is associated with activated/effector phenotypes in T cells

**Figure 3:**
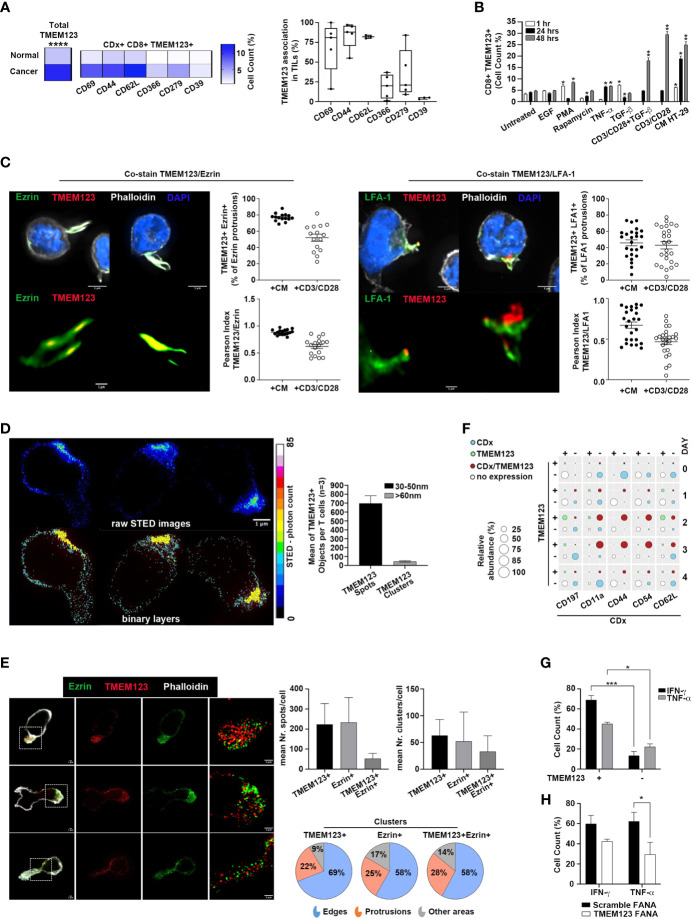
TMEM123 is induced by TCR activation and by cancer cells conditioned medium and is associated to effector T cell phenotype. **(A)** TMEM123 is co-expressed with activation markers in TILs. Co-expression analysis of TMEM123 in tumour infiltrating CD8+ T lymphocytes with the indicated markers. Heat-maps show the mean of 4 independent experiments. Graph shows the percentage of association of TMEM123 with each marker, in CD8+ T cells. **(B)** TMEM123 is induced by microenvironmental stimuli. PBMC from healthy donors were incubated with listed stimuli for 1h (white), 24h (black) and 48h (grey) and the presence of TMEM123 on the surface of CD8+ T cells was followed by FACS analysis, normalizing on the untreated samples. **(C)** TMEM123 distribution in Jurkat cells after stimulation with CM or CD3/CD28. Representative images at 100x magnification in confocal microscopy, where cells are shown with all channels in upper row, and with highlighted zoomed-in magnification of protrusions with evident localization of both Ezrin (green) or LFA-1 (green) and TMEM123 (red) in lower row. Cellular actin protrusions were quantified for each cell in all FOVs and dot-plots show the quantification of TMEM123+ Ezrin+ or TMEM123+ LFA-1+ protrusions in percentage towards Ezrin+ or LFA-1 protrusions (upper dot-plots), in which specifically the co-localization of both fluorophore signal was quantified in terms of Pearson Index (lower dot-plots). **(D)** TMEM123 nanoscale distribution in CD3 T cells after stimulation with CM and seeded on poly-lysine coating. Cells were immunolabelled for TMEM123 followed by secondary immunolabelling fluorescently conjugated with StarRED to super-resolve images with 775nm STED deletion. Three representative cells were shown in raw STED images (upper images) and in classified binary layers (lower images) to evaluate the distribution of intracellular TMEM123 spots (cyan; dimensional range 30-50nm) and clusters (yellow; dimensional range >60nm). Numbers of single TMEM123 spots (black bar) and TMEM123 clusters of molecules (grey bar) were quantified per cells in bar-chart. **(E)** A total of n=52 cells were analysed by STED microscopy to observe and correctly localize spatially TMEM123 immuno-labelled molecules (StarRED fluorophore, red LUT in images) relative to Ezrin, immuno-labelled as well (StarORANGE fluorophore, green LUT in images). The bar graphs show the quantification for single protein spots/cell (left) and for protein clusters/cell (right). The pie charts represent the proportion of clusters in cell edges, protrusions or all other dispersed cell areas. **(F)** TMEM123 is co-expressed with known T lymphocytes markers highly enriched in T cell adhesion and migration. Dot plot represents FACS analysis on CD8+ T cells for TMEM123 expression as standalone or in co-expression with tested markers. Data represent the average of at least 3 experiments. **(G)** TMEM123 positive CD8 T cells express high level of effector cytokines. CD3/CD28 activated CD8 T cells from PBMC of HD were treated with PMA-ionomycin-brefeldin-A and stained for indicated cytokines. Graph reports the percentage of cytokine produced from TMEM positive or TMEM negative T cells as determined by FACS analysis. **(H)** TMEM123 silencing impairs production of effector cytokines. CD3/CD28 activated CD8 T cells were further treated with TMEM123-FANA and cytokine expression was assessed by FACS. Statistical significance is denoted by asterisks (p-value *= <0.05; **= <0.01; *** = < 0.001; **** = < 0.0001).

We focused our interest on TMEM123 expression in tumor-infiltrating CD8+ T lymphocytes. To understand whether TMEM123 might contribute to the anti-tumoral effect of CD8+ T cells, we investigated the activated or dysfunctional state of TMEM123 positive cells, *via* co-expression analysis with a subset of lymphocyte state markers, indicative of either activation or suppression/exhaustion. Interestingly, we observed that in CRC-infiltrating CD8+ T cells, TMEM123 was specifically co-expressed with the activation markers CD69, CD44 and CD62L. Conversely, a low association was observed with the suppression/exhaustion markers TIM-3 and CD39, and an intermediate level of association with PD-1 (**Figure 3A**). Similar results were also obtained by analysing the fraction TMEM123+CD4+ cells (**Figure S3A**). Collectively, these results indicate that TMEM123 expression is positively associated with activated lymphocyte effector functions. Next, we tested whether the expression of TMEM123 responded to T-cell receptor (TCR) activation *via* anti-CD3/CD28 T cell activation and to tumor microenvironmental stimuli known to affect fates and intrinsic pathways of T lymphocytes, relevant for trafficking, differentiation and function. PBMC from healthy donors were incubated with TNFα, TGFβ, rapamycin, PMA, EGF or with the conditioned media (CM) of cancer cells (HT-29), containing several TME factors, including cytokines, growth factors and chemokines. The presence of TMEM123 was monitored in CD8+ (**Figure 3B**) and CD4+ (**Figure S3B**) T cells by FACS analysis. In both CD8+ and CD4+ T cells, TMEM123 expression significantly increased on the cell surface over time upon CD3/CD28 activation and/or exposure to CM. In CD8+ T cells, TMEM123 also increased in response to the mitogenic stimuli, PMA, TNFα and TGFβ treatments, while in CD4+ T cells the response was evident following EGF and PMA, and to TGFβ (**Figures 3B** and **S3B**). In addition, TMEM123 levels were also upregulated in CD8+ and CD4+ T cells isolated from PBMC and from matched colon tissues of CRC patients co-cultured with HT-29 cells (ratio 10:1). Such upregulation was more marked in CD8+ than in CD4+T cells (fold increase of 4, 7 and 2, respectively in CD8+ T cell of PBMC, normal colon and CRC; average values of 3 clinical samples) (**Figure S3C**). Since expression of TMEM123 in blood T cells is very low, stimulation with CD3/CD28 or CM was used as initial experimental step in all experiments requiring the identification and specific selection of TMEM123+ peripheral T cells.

We monitored the kinetics of TMEM123 expression in peripheral CD8+ and CD4+ T lymphocytes of healthy donors over four days of CD3/CD28 stimulation and we found that TMEM123 was significantly induced, with a peak at days 2-3 (60-70% of positive cells) (**Figure S4A**). Nevertheless, TMEM123 is not an essential factor of T cell activation as its specific silencing, with FANA-aso oligonucleotides (which are spontaneously captured by primary cells without transfection reagents or other stress conditions) (15) efficiently reduced the protein expression but did not alter the overall T cells activation state, as measured by CD69 and CD62L markers (**Figure S4B**). The specific set-up of efficient silencing was performed in T cells, and confirmed in Jurkat cells, known to express TMEM123 endogenously (10, 11), and used as model (**Figures S4C-F**). Intriguingly, by confocal microscopy on Jurkat cells stimulated with CM or CD3/CD28, we observed an accumulation of TMEM123 clearly localized in cell protrusions (**Figure 3C**), suggesting a possible implication of TMEM123 in cell adhesion and migration. Localization of TMEM123 was assessed by the association of Ezrin (16), a canonical uropod marker or with LFA-1 (17) an integrin that plays a critical role in the regulation of adhesion and de-adhesion of immune cells. TMEM123+ Ezrin+ cellular protrusions represented a significant fraction of the total Ezrin+ protrusions (respectively 77% ± 1,2% after CM and 52% ± 4.3% after CD3/CD28), assessed by co-localization of both fluorophore signals (Pearson Index, respectively 0.9 ± 0.01 after CM and 0.6 ± 0.04 after CD3/CD28) (**Figures 3C**, **S5A-C**). Analyzing the LFA1+ protrusions, we found a fraction also positive for TMEM123 (respectively 46% ± 3.6% after CM and 43% ± 4.5% after CD3/CD28), assessed by co-localization of both fluorophore signals (Pearson Index, respectively 0.7 ± 0.04 after CM and 0.5 ± 0.03 after CD3/CD28), less pronounced compared to the Ezrin+ fraction (**Figures 3C**, **S5B**). To further investigate the distribution of TMEM123 nanoscale organization in CD3 T cells after stimulation with tumor CM, we used super-resolution STED microscopy. With a spatial resolution of ∼30nm, STED resolved individual TMEM123 spots packed at different densities. TMEM123 spot densities was much higher in the region of the membrane forming protrusions or in areas of adhesion to the glass-slide. We measured the fluorescence intensity of individual TMEM123 spots (defined as molecule-aggregates, having a diameter ranged 30-50nm) and of TMEM123 clusters (defined as spot-aggregates, having a diameter in the range of 60-120nm) (**Figure 3D**). By analysing the relative localization of TMEM123 towards Ezrin, we found that these proteins share similar spatial localizations within CD3 T cell area, often at one particular cellular edge, but display only minimal direct co-localization, implying cellular proximity but no direct structural protein interaction, at the level of 30nm resolution (**Figures 3E**, **S5D**). To better represent the spatial proximity among clusters of TMEM123+ and Ezrin+, a distribution of clusters over the cellular area was elaborated using the mathematically derived coordinates of fluorophore spots in the analysed fields of view (**Figure S5E**). The pie chart represents the analysis of the proportion of clusters in cell edges, cell protrusions or all other dispersed cell areas. We observed a clear-cut difference, with high percentage of both single positive and double positive clusters in cell edges (58-69%) or protrusions (22-28%) (**Figure 3E**). Based on these observations, we monitored co-expression of TMEM123 with other known proteins involved in T cell adhesion and migration (LFA-1-CD11a subunit, ICAM-1-CD54, CD44, L-Selectin-CD62L, and CCR7-CD197) (18) on both CD8 (**Figure 3F**) and CD4 (**Figure S6A**) lymphocytes. As a trend, TMEM123 was concomitantly expressed with ICAM-1 at days 2 – 3, but returned to baseline on day 4 from activation, while ICAM-1 levels still persisted. A high fraction of cells co-expressed TMEM123 with CD62L, CD11a, CD44, although these proteins appeared earlier on the cell surface than TMEM123. In addition, we compared secretion of IFNγ and TNFα in stimulated TMEM123+ versus TMEM123- T cells. A significant fraction of TMEM123+ CD8+ and CD4+ T cells mainly produced IFNγ, TNFα, while TMEM123- T cells showed a much lower production of these cytokines (**Figures 3G**, **S6B**). In line with these results, TMEM123 silencing reduced cytokine release in TMEM123+ CD8+ (**Figure 3H**) and CD4+ T (**Figure S6C**) cells, with a more marked effect on TNFα.

Taken together these data indicate a potential role for TMEM123 expression in T cell effector functions, including adhesion and activation.

### TMEM123 is involved in T lymphocytes motility, chemotaxis and trans-endothelial migration

**Figure 4:**
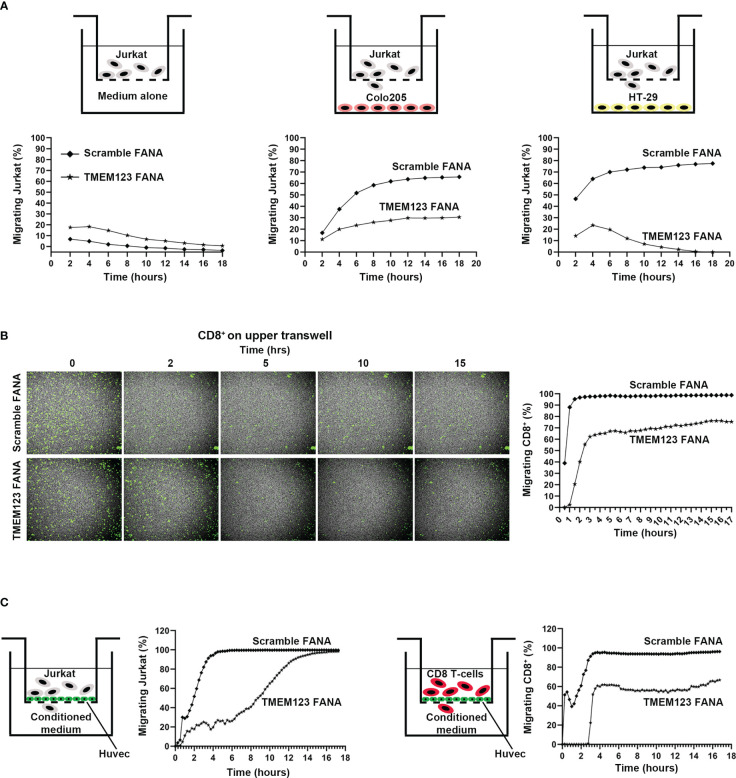
TMEM123 promotes chemotactic and trans-endothelial migration of T lymphocytes. **(A, B)** TMEM123 promotes migration of Jurkat and CD8 T cells. In **(A)** Boyden assay for CFSE-labelled Jurkat cells: the drawings schematize the set-up used for the assay; graphs represent the percentage in migrating cells. In **(B)** Boyden assay for CFSE-labelled activated CD8+ T cells: representative microscopic images show TMEM123 positive cells in the upper chamber during the acquisition time frame. Graph shows the percentage in migrating cells. **(C)** TMEM123 promotes trans-endothelial migration of Jurkat and CD8 T cells. Jurkat (left panels) or activated CD8 T cells (right panels) were suspended into the upper chamber of a Boyden chamber. Monolayer of HUVEC were seeded on the semi-permeable membrane of the chamber. Graph represent trans-endothelial migration in tested conditions. TMEM123 silencing remarkably impaired cells migration in all analysed conditions.

Intrigued by TMEM123 co-expression with proteins involved in leukocyte migration, chemotaxis and endothelial transmigration, we investigated the role of TMEM123 in these functions *via* Boyden *in vitro* assays and live monitoring of T cell migration, over 18h using spinning disk confocal microscopy. We first monitored migration of TMEM123-silenced CFSE-labelled Jurkat or control Jurkat (scramble) cells, as suitable cellular model endogenously expressing TMEM123, to recapitulate T cells migration (19). The cells are seeded in serum-free culture medium towards the Boyden lower chamber containing 10% FBS- RPMI medium, Colo205 or HT-29 cells. The factors secreted from cells in culture medium include metabolites, cytokines, growth factors and chemokines which play a key role in lymphocytes recruitment, as well as in inducing chemotaxis through the activation of G-protein-coupled receptors (20). Jurkat cells rapidly migrated towards wells containing either HT-29 or Colo205 cells, compared to a significantly slower migration towards FBS-RPMI medium. TMEM123 silencing in Jurkat cells remarkably impaired cells migration in all analyzed conditions (**Figure 4A**). A similar migration phenotype was observed using CD8+ T cells isolated from healthy donors’ PBMC. Specifically, TMEM123 silencing decreased CD8+ T cells migration towards the lower chamber containing either HT-29 CM (**Figure 4B**) or Colo205 CM (data not shown). Having shown such role of TMEM123 in T cell chemoattracted migration, we further tested whether TMEM123 might be involved in T cells diapedesis, as already described for ICAM-1 and LFA-1 (21). Jurkat and CD8+ T cells were tested for the ability to cross a tight monolayer of HUVEC endothelial cells grown to confluence in Boyden chamber. Relevantly, both cell types migrated quickly through the HUVEC monolayer, whereas TMEM123 knock-down caused a significant reduction of trans-endothelial migration (**Figure 4C**).

### Reduction of TMEM123 expression alters cytoskeleton organization and downstream signaling pathways

**Figure 5:**
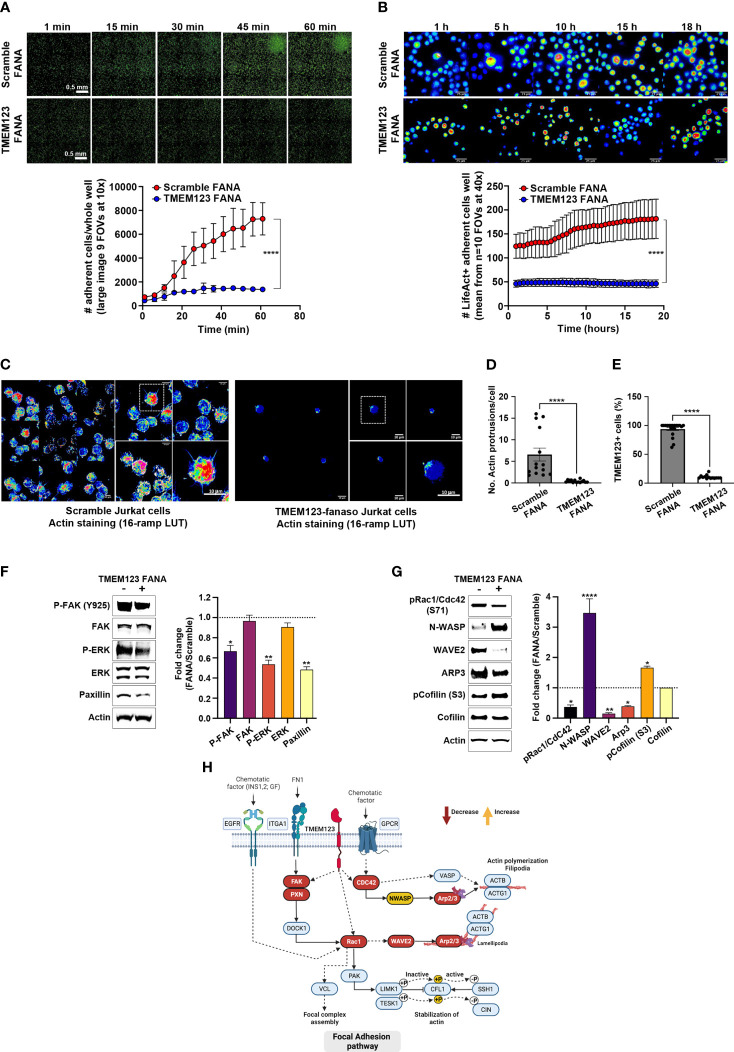
TMEM123 is a molecular regulator of the actin cytoskeleton dynamics pathway. **(A, B)** TMEM123 is involved in cell adherence. Upper panels: Representative images of time-recordings of Jurkat cells adherence, visualized *via* life-actin live-stain, following 48h incubation either with scramble FANA or with specific TMEM123 FANA. The images were acquired as whole sample **(A)** in large image mosaic of 9 FOVs at 10x magnification or in random FOVs at 40x magnification for better detail visualization **(B)**. The graphs in the lower panels, show the image quantification of adherent life-act positive Jurkat cells per FOV at specific time points. N=4 biological samples/condition, n=10 technical replicates. ****= p-value< 0.0001, unpaired Mann-Withney test. **(C–E)** TMEM123 silencing alters actin morphology. **(C)** Representative images of best focal plans at 40x and 100x magnification from Jurkat cells stained with phalloidin for actin morphology following 48h incubation either with scramble FANA (left) or with specific TMEM123 FANA (right). **(D, E)** The graphs show a quantification of the number of actin protrusions and counts of TMEM123+ cells (90%). N= 867 cells analyzed over n=34 FOVs. **= p-value< 0.01, (ns)= not significant, unpaired Mann-Withney test. **(F, G)** TMEM123 silencing affects the actin cytoskeleton signaling. CD8 T cells were TMEM123 silenced by treating the cells with TMEM123 specific FANA and cell lysates was immunoblotted with antibodies against the indicated proteins. The densitometry value of each band was determined with ImageJ and normalized to β-actin. Data was presented as mean ± SE of three independent experiments. *=p-value<0.05, **=p-value<0.01, ****=p-value<0.0001. The densitometry values in the histogram are expressed as fold changes relative to scramble FANA, which was assigned a value of 1 (dotted line). **(H)** Proposed model of the “regulation of actin cytoskeleton” by TMEM123 was adapted from KEGG: Kyoto Encyclopedia of Genes and Genomes and created in Biorender.com. Solid arrows represent molecular interaction or relation; dash arrows represent indirect link or unknown reaction.

We then examined whether the T cell migration/adhesion defect due to TMEM123 silencing might cause substantial changes in overall cell morphology. We first evaluated the adhesion efficacy of Jurkat cells over time following 48 hours treatment with scramble FANA or with specific TMEM123 FANA. The impairment of adhesion in TMEM123 silenced cells to poly-L-lysine-coated 96-well optical plate was already evident in the first 15-30 minutes of live imaging (**Figure 5A**). Such adhesion defective phenotype was linked to huge loss in the formation of actin filaments, as assessed by image quantitative analysis and expressed as numbers of adherent Lifeact positive Jurkat cells per FOV (n=10 40X) (**Figure 5B**). Jurkat cells were also stained with phalloidin and imaged by confocal microscopy at the best focal plans for Actin marker (**Figure 5C**) highlighting again a defective adhesion capacity accompanied by a significant loss in the number of actin protrusions following TMEM123-silencing (**Figure 5D**), as evaluated by double staining with TMEM123+ (**Figure 5E**). Furthermore, we investigated the cellular mechanisms underlying the morphological and functional changes observed following the silencing of TMEM123. We first analyzed in CD8+ T cells the protein levels of Focal Adhesion kinase (FAK), scaffolding component of FA and Paxillin, anchoring protein, together with ERK, which regulates cell motility through FAK and Paxillin phosphorylation (22). TMEM123 silencing significantly reduced both paxillin levels and phosphorylation of FAK and ERK1/2 (**Figure 5F**), thus supporting the relevance of TMEM123 in cytoskeleton arrangement and motility.

As to better define the molecular changes underlying the cytoskeleton remodeling, following the partial loss of TMEM123, we monitored several signaling proteins such as the Rho GTPase Rac1/Cdc42 and their downstream effectors linking to cytoskeleton actin, such as the members of the Wiskott-Aldrich syndrome protein (WASp) family, including WASp, the WASp family verprolin-homologous protein-2 (WAVE2), and the Arp2/3 functional complex, which is activated by the former two proteins (23–25). Upon TMEM123 silencing in CD8 T cells we observed a reduced phosphorylation of Rac1/Cdc42, indicative of an inactive conformation, together with a marked downregulation of WAVE2 and Arp2/3 complex, as judged by Arp3 reduction, whilst an up-regulation of N-WASp (**Figure 5G**). When assessing Cofilin, potent regulator of actin filament dynamics (26), TMEM123 silencing induced an increase of the phosphorylation at serine-3, known to be associated to an inactive state (**Figure 5G**).

Collectively, our results demonstrate that TMEM123 is a molecular modulator of actin cytoskeleton dynamics, and its reduced expression causes severe defects in T lymphocytes migration complexes, as schematized in the signaling pathway model proposed in **Figure 5H**.

### TMEM123 expression is pivotal for TILs migration towards and clustering upon cancer cells

**Figure 6:**
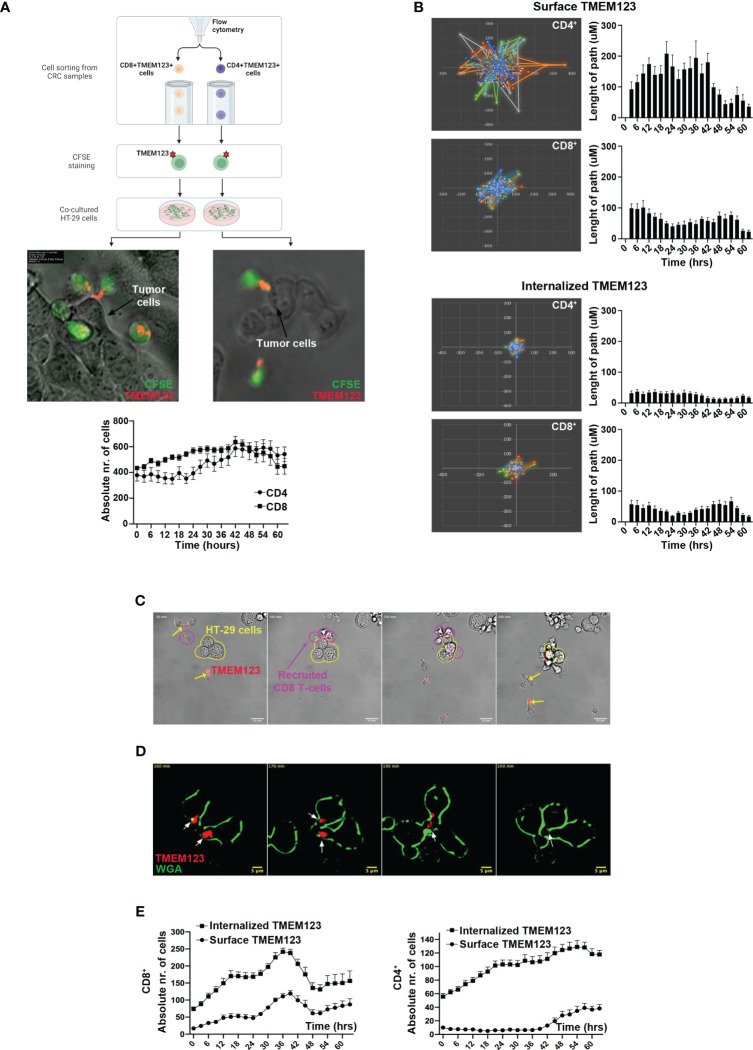
TMEM123 localizes at the clustering sites of TILs towards cancer cells and is essential for T cell motility. **(A)** Scheme of experimental procedure, representative images and derived quantifications from time-lapse analysis of cell-cell interactions and TMEM localization in CD4 and CD8 cells. Images were selected as time-frame snapshots, highlighting TMEM123 localization at T cell uropod-like protrusions. Graph shows the counts of TMEM positive CD4 (black circles) and CD8 (black squares) T cells over the time of imaging. **(B)** Polar plots over field of view and histograms over time show the spatial movement, in terms of movement display and path-length respectively, of positive TMEM123 CD4 (upper graphs) and CD8 (lower graphs) T cells with either TMEM 123 on the surface or internalized TMEM123. **(C–E)** Live-occurring cellular internalization of TMEM123 is observed within the cytoplasm of the T cells, following stable encounter with cancer cells. **(C)** Time frame snapshots showing TMEM123+ CD8 T cells (magenta contour) recruited towards a cluster of cancer cells (HT29) (contoured in yellow polymorphic shape). Yellow arrows point to anti-TMEM positive rafts on T cell uropods. **(D)** High resolution dark-field contrasted time frames. In red anti-TMEM123 mAbs (indicated by white arrows) and in green T cells membrane (WGA). **(E)** Graphs show numeric quantifications of surface (circles) and TMEM123-internalized (squares) CD8 (upper) and CD4 (lower) T cells over the time of imaging.

As data indicate important effects of TMEM123 on the migration of T cells, we further investigated the interplay between TMEM123+ TILs and cancer cells in co-culture assays. In brief, CFSE-labelled CD4+ and CD8+ T cells sorted ex vivo from CRC samples were live-stained with stably fluorescently conjugated anti-TMEM123 antibody (Qdot-705) and afterwards incubated with HT-29 cells grown to pre-confluency and monitored over a 2-day recording of 3-hour time-lapse experiment (**Figure 6A**). TMEM123 showed a polarized distribution both on CD4+ and CD8+ T cells, often focused on cell protrusions at one edge of the cell (**Figure 6A**, **Video S1**), likely the uropod structure. TMEM123 was particularly abundant on the surface of those T cells adhering to and invading through HT29 cell groups (**Figure 6A**), well visible at cell clustering sites (**Video S2**), and often localized in hotspots of T cell anchoring. In the first half of the monitoring period, the absolute number of TMEM123+ CD8+T cells interacting with cancer cells was slightly higher than TMEM123+ CD4+ T cells (up to 1.6-fold at 24h), whereas afterwards both cell types reached a plateau with comparable numbers (**Figure 6A** bottom graph). The chosen time-lapse of recording (3h-loop), selected for best feasibility over long imaging condition (60h) was not suitable to analyze speed of T cell movement. Therefore, we monitored the influence of TMEM123 surface labelling on T cell motility phenotype by analyzing the trajectories and length of walking paths of CD8+ and CD4+ T cells (approximately 1400 independent cell trajectories globally measured in 9 fields of view/cell type). We found that TMEM123+ CD4+ T cells showed a more pronounced and scattered motility than TMEM123+ CD8+ T cells (up to 2-fold longer length of walking path, on average) with slight fluctuations over time (**Figure 6B**). In both T cell types, absence of TMEM123 surface expression was associated with a significant decrease of cell displacement and parallel increase of on-the-spot confinement (**Figure 6B**). TMEM123 labelling tended to disappear from T cell surface soon after their detachment from cancer cells, as TMEM123-labelled protein was internalized in the cytosol (**Figures 6C, D**; **Video S3** and **S4**), a process that appeared to be slightly faster in CD8+ than in CD4+ T cells (**Figure 6E**). All together, these data might indicate a role for T cell TMEM123 surface expression in directing migration and cell adhesion, prior to T cell confinement and activation.

### TMEM123 drives migration and clustering of CD8+T lymphocytes in tumor organoids and promotes killing of cancer cells

**Figure 7:**
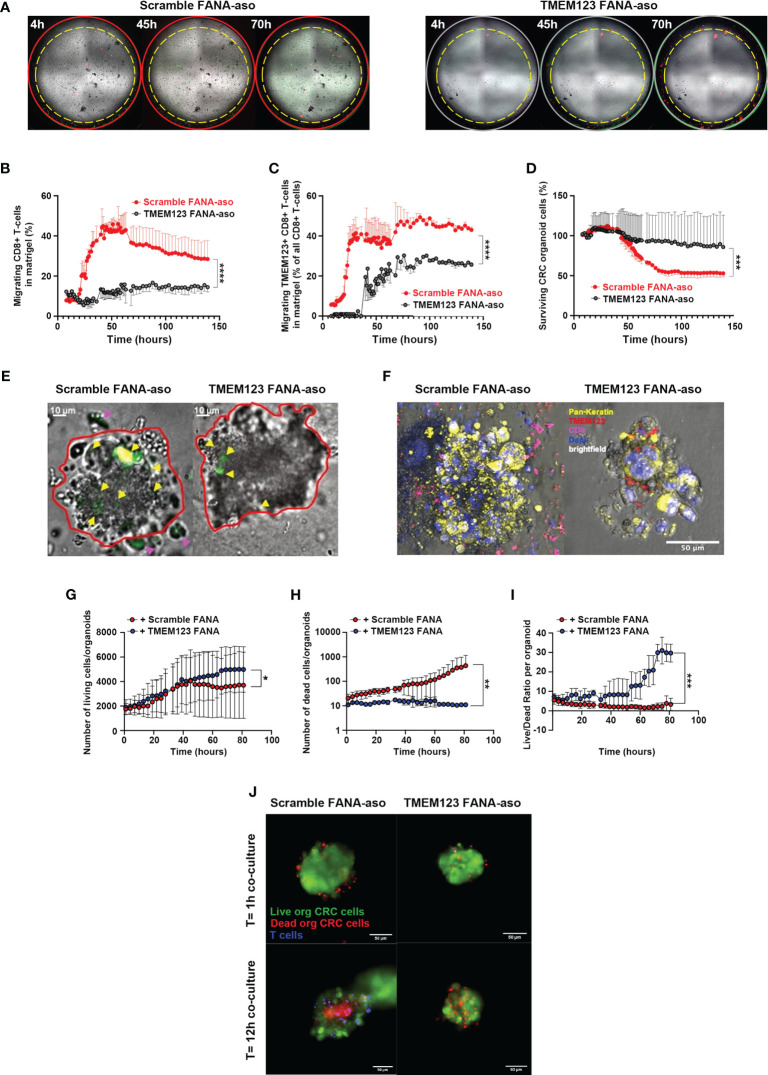
TMEM123 promotes CD8 T cells clustering on cancer cells in CRC-tumoroid co-culture models. TMEM123 silencing affects migration of CD8 T cells in co-culture with tumoroids. **(A)** Representative low magnification whole-well images of co-cultures (CD8 in green; CD8+TMEM123 in green+red). **(B)** CD8 migration was monitored and plotted over time. **(C)** TMEM123+ CD8 T cell migration was analyzed comparing scramble FANA-aso versus TMEM123 FANA-aso T cells (p<0.0001, 2-way ANOVA; n=4). **(D)** CRC-tumoroid cell survival during imaging comparing the co-culture with scramble FANA-aso CD8 T cells versus TMEM123 FANA-aso CD8 T cells (p<0.0001, 2-way-ANOVA; n=4). **(E)** Representative high magnification images show static frames at 140 hours of co-culture between CRC organoid (red binary contour) and either scramble FANA-aso CD8 T cells (left) or TMEM123 FANA-aso silenced CD8 Tcells. T cells already migrated within CRC-organoids are highlighted by yellow arrowheads, whereas T cells still migrating towards the organoid (even after 140 h) are highlighted by magenta arrowheads. **(F)** Representative images show whole-mount staining for TMEM123 (red), Pan-Keratin (yellow), CD8 (magenta) of fixed CRC organoids at the end of the live co-culture experiments monitored in **(A–E)**. **(G–I)** Analysis of the CRC-organoids live-stained for live/dead labelling during the co-culture with CD8 T cells. A total of n=121 CRC-organoids and n=61430 T cells was analyzed over time. Quantification *via* digital segmentation and object classification shows the number of alive cells **(G)** and dead cells **(H)** within the organoid structures over time, also plotted as live/dead cellular ratio normalized for number of organoids **(I)**. **(J)** Representative image of the co-culture among CRC-organoids and T cells with or without TMEM123 silencing. CRC organoids live-stained for live/dead labelling (respectively green and red) and T cells live-nuclearly labelled with live-hoechst solution. Magnification 10X, n=3 selected FOVs for each well. Mann-Whitney unpaired comparisons and Kruskal-Wallis tests for non-parametric variable analysis over time. *= p-value < 0.05, **= p-value < 0.01, ***= p-value < 0.001, ****= p-value < 0.0001.

As to better confirm TMEM123 role in CD8+ T cell migration in a more complex milieu, mimicking cancer microenvironment, we employed a human tumor organoid model, better simulating architecture of cancer tissues than cancer cell line monolayers. CRC tumoroids were generated from crypts of CRC biopsies seeded on a matrigel drop and grown for 2-3 weeks, to obtain structurally mature tumoroids. Matched CFSE-labelled TMEM123+CD8+ T lymphocytes of CRC patients (n=2) were treated with TMEM123-FANA or a scramble FANA control. Cells were then labelled with stably fluorescent anti-TMEM123 antibody and co-cultured with CRC organoids, grown in Matrigel drops over optical culture 96-well plates. We monitor T cell migration for seven days by live image spinning-disk confocal microscopy (**Figure 7**). Analysis showed that TMEM123 silencing reduced migration capability of CD8+ T cells towards CRC organoids (**Figures 7A–C**). In addition, the presence of TMEM123+ CD8+ T cells induced a reduction in organoid cell numbers, as assessed by quantification of organoid cell counts over time, normalized to the counts at the initial time point (**Figure 7D**). Conversely, organoid cell counts were almost unaltered when co-cultured with TMEM123 silenced CD8+T cells (**Figure 7D**). Moreover, T cell clusters enriched in TMEM123+ CD8 T cells showed a specific directional movement towards the organoid (visible one specific directional track of T cell movement in green, towards the red-countered organoid structure in **Video S5**) with numerous CD8+ T cells visible within the organoid mass and attached to the organoid edges (**Figure 7E**). Whole-mount fixation of 3D samples and immune-staining at the end of live co-culturing confirmed such organoid-T cell interactions and highlighted TMEM123 protein expression also at the level of CRC organoid-forming cells (**Figure 7F**). In another set of experiments, we measured the effect of TMEM123 silenced or not CD8+T cells on tumoroid killing using a life/death staining suitable for live-imaging recordings. Quantification shows the number of both alive cells (**Figure 7G**) and dead cells (**Figure 7H**) within the organoid structures over time, also plotted as live/dead cellular ratio normalized for number of organoids (**Figure 7I**). **Figure 7J** shows a representative image during the acquisition. As internal controls, tumoroids without T cell co-culture were seeded on other wells in the same optical well-plates throughout the co-culture experiment and analyzed *via* whole-mount staining for morphological control (**Figure S7**). Overall, these data further confirm that TMEM123 drives T cell migration and clustering and contributes to the killing activity of CD8+ T lymphocytes on cancer cells.

## Discussion

It is becoming increasingly clear that cancer progression is influenced by the continuous interaction of cancer cells with immune cells of the tumor microenvironment. Both CD8+ and CD4+T cells can mount responses against many human cancer types, especially those with higher mutational burden (3, 27) and several studies in different tumour types have shown a correlation between tumor-infiltrating effector CD8+ T cells and favorable clinical outcomes (1) (28). However, the anti-tumor activity of these cells is partially inhibited by immunological checkpoints, such as the PD-1/PD-L1 interaction axis, and other immune cells, such as regulatory T cells, M2-macrophages and a number of other factors that induce a prevailing immunosuppressive state (1), favoring the tumor.

In CRC, the impact of infiltrating cytotoxic CD8+ and Th1 CD4+ T lymphocytes within the TME on containing the growth of established colorectal cancer and limiting metastasis is well documented. For example, microsatellite instability high (MSI-H) colorectal cancer has a higher concentration of CD8+ cytotoxic and Th1 CD4+ T cells than MSS (microsatellite stable) cancers, contributing to the better prognosis of these cancers (29, 30). The molecular factors contributing to the effector function of TILs are only partially known.

This study highlights human TMEM123 (alias Porimin) as a key immunosurveillance element in CRC, expressed in TILs and associated with their effector activities. By IHC analysis of clinical samples we showed that TMEM123 is moderately expressed in CRC tissue samples, but it is absent in adjacent non-malignant epithelial tissues. Interestingly, TMEM123 is clearly expressed in tumor-infiltrating immune cells of the same cancer tissues, independent of its expression in cancer cells, suggesting that expression of TMEM123 is not intertwined in the two compartments.

This evidence might indicate that, the presence of TMEM123 is associated to a common feature of these cells, possibly linked to a very activated state or a migratory phenotype. TMEM123 expression was found both at the level of the plasma membrane and in the cytosol, likely indicating its involvement in membrane trafficking in the endocytic pathway. This hypothesis is also supported by the presence of a lysosome/endosome targeting YXXφ motif and by the internalization propensity observed in T lymphocytes, reported in this study. In this study we decided to primarily focus our attention on tumor-infiltrating T lymphocytes expressing TMEM123. *In situ* immunofluorescence and/or FACS analyses of clinical samples from CRC patients revealed that TMEM123 is mostly enriched in CD8+ and CD4+ T cells of the immune infiltrates present in primary and metastatic cancers, being simultaneously expressed in synchronous and metachronous metastasis, while it is expressed at much lower level in T cells resident in proximal normal tissues, and almost negative in blood-circulating T cells and in non-activated or exhausted T lymphocytes. Remarkably, the presence of TMEM123 in intra-tumoral CD8+ positively correlated with the overall and/or the metastasis-free patients’ survival, suggesting that it can provide a physiologic advantage to patients. Furthermore, both *in vitro* CD3/CD28 activation and incubation with cancer cells conditioned medium, was enough to induce the expression of TMEM123 in peripheral T cells. Remarkably, high and super-resolution microscopy analysis showed that TMEM123 accumulates in T cell protrusions, also including the uropod, a posterior appendage of polarized lymphocytes serving for motility and migration (31), in concerted localized co-expression with other T cell markers involved in cell motility, such as Ezrin and LFA-1.

This study also shows that TMEM123 is important for T cell adhesion, chemotaxis and trans-endothelial migration, as assessed by transmigration *in vitro* assays, where we observed an active migration of TMEM123+ CD8+T lymphocytes towards soluble factors present in cancer cells conditioned medium, known to be enriched both in growth factor and in migration-inducing chemokines, which was strongly inhibited by TMEM123 silencing. Based on our data, TMEM123 could have a specific role in the recruitment of T cells to neoplastic sites in response to chemotactic stimuli. In addition, this protein takes part to the TCR-induced adhesion process by facilitating crosstalk between the TCR and the actin cytoskeleton network to induce contractile forces. We observed that TMEM123 silencing reduces phosphorylation of FAK at the Y925 residue located in the FAT domain of the protein. FAK is a non-receptor tyrosine kinase, acting as broad regulator of cell morphology and motility, influencing cytoskeleton and actin polymerization. Depending on the cellular context, FAK phosphorylation at Tyr-925 acts as a molecular switch coordinating either focal adhesions disassembly or the formation of a cell edge protuberance, modulating cell migration and cell protrusion28. In lymphocytes, FAK is phosphorylated downstream of TCR signaling or other co-stimulatory and cytokine/chemokine receptors, and also by adhesion receptors (32). For instance, integrin LFA-1 (bound to its ligand ICAM-1) directly acts on FAK, thus inducing the remodeling of T lymphocyte morphology (33).

Reduction of TMEM123 causes a profound impairment of the actin cytoskeleton organization in CD8 T cells, which consequently results in a defective cell migration machinery. The molecular inactivation cascade is signaled through Rac1/Cdc42 to WAVE2 and the Arp2/3 complex. The WAVE complex localizes at the edge of lamellipodia, specific protrusions formed during migration. The actin filaments that form lamellipodial networks are mostly generated through nucleation or branching effected by Arp2/3 complex (23–25). Since migratory lymphocytes adopt a polarized cell form defined by the formation of a lamellipodium at the anterior end and a uropod at the back of the cell, a decrease of WAVE2 and Arp2/3 complex results in remarkable effects on the migration mode of T cells to and within tumor sites (34, 35).

Unexpectedly, in the CD8 T cells only we observed an up-regulation of N-WASp (WASp ubiquitously expressed). This effect might be due to the decrease in Cdc42, which upon cells stimulation, binds to the WASp GBD domain, thereby releasing WASp from its auto-inhibitory conformation and exposing the VCA domain which binds to the Arp2/3 complex (36, 37). Therefore, partial loss of regulation of Cdc42 activation possibly results in an accumulation of the N-WASp protein within the T cell. Furthermore, TMEM123 silencing correlated with an increase in serine-3 phosphorylation of Cofilin, a protein with key role in maintaining and extending the lamellipodial protrusion at the leading edge of migrating cells. The level of Cofilin phosphorylation in cells is critical for the regulation of actin cytoskeleton dynamics and morphological changes. Phosphorylated cofilin is unable to bind actin filaments and actin monomers (26, 38).

Besides modulating T cell cytoskeleton rearrangements, TMEM123 also contributes to the cytotoxic functions of CD8+ T-cells, since its silencing causes a reduction of expression of Th1 effector cytokines, mainly TNFα but also IFN-γ, and IL-2. It is known that in T cells, cytokine expression, motility and cytoskeleton organization are intertwined in a dynamic process, since cytokine secretion promotes T cell migration (33) and, on the other side cytoskeleton organization influences T cell activation, adhesion and migration (39). In this entangles context, due to its role in cytoskeleton organization TMEM123 might indirectly influence cytokine production.

Finally, our study provides novel evidence on the role of TMEM123 in the interaction between effector T cells and cancer cells. By co-culturing experiments, we found that TMEM123 localizes in anchoring sites of CD8+ T cells attacking the cancer cells and forming clusters of lymphocytes on their surface, indicating that TMEM123 plays an important role in TILs adhesion to cancer cells. In line with this, co-cultures of TMEM123+ CD8+ T lymphocytes with CRC tumoroids further demonstrated that TMEM123 expression is required for the directional migration of CD8+T cells towards the organoid, which is pivotal for further attacking and eliminating the cancer cells. Dedicated studies are required to compare the tumoroid morphology during co-cultures with TMEM123 positive/silenced CD8 T cells.

Overall, this study elucidates the crucial role of TMEM123 in CRC microenvironment, by acting as a cell surface regulator for the adhesion and migration of T lymphocytes. As such, we hypothesize that this protein may serve as sensor of tumor microenvironmental stimuli in T cells, likely also due to its high glycosylation state. Indeed, protein glycosylation is well-known to play a role in regulation of cell adhesion, between leukocytes and vascular endothelial cells. Moreover, glycans are also involved in the modulation of the immune response with glycan binding proteins, such as siglecs and galectins (40). In addition, we believe that TMEM123 may be an attractive target for CD8+ based immunotherapy, for the design of molecular agonists able to promote the recruitment of T cells to the tumor site and attack of cancer cells, mediating anti-cancer Th1 immune responses. To this aim, an important future objective is the identification of TMEM123 interaction network, as to better decipher TMEM123 role in the balance between killing and outgrowth of cancer cells. Last but not least, our study provides the rationale of strengthening TIL-associated TMEM123 as a prognostic factor in CRC, as evident by the better survival rate of patients with increased TMEM123+ TILs.

This study has a number of limitations. A limitation is that our findings were not further investigate in mouse models, such as by passive transfer experiments of TMEM123+ T cells or knockout mouse model for TMEM123. Indeed, the TMEM123 mouse ortholog shows only 54-56% amino acid identity with the highest similarity in the C terminal part and lowest one identity in the external N terminal region exposed to the TME. Consequently, it is not clear whether mouse cancer models may represent a valid model to study TMEM123 function in the TME. In addition, mouse studies would require the use of a high number of animals, which we believe would not be ethically justifiable, for the purpose of the study. Future studies using reagent materials raised against TMEM123 mouse ortholog will be done to assess whether the mouse is an appropriate model to study TMEM123 function in the TME. At present, the absence of a proof-of-principle animal model pushed us to propose the tridimensional tumoroid models to test the anti-cancer cytotoxic activity of TMEM123+ CD8 T lymphocytes. Organoids and tumoroids are now wildly recognized to partly reproduce the human context in 3D *in vitro* architecture and they can be easily employed for co-cultures and mechanistic studies. We believe these models are suitable to appraise the crucial role of TMEM123 in TILs and explain its positive correlation with patients’ survival. Indeed, we observed a clear effect of TMEM123 in helping the directed migration and adhesion of lymphocytes to tumoroids, finally leading to efficient killing of cancer cells in a 3D context. Another limitation is that we did not investigate the specific molecular component/s of the TME is able to upregulate TMEM123. Our hypothesis is that cytokines stimulating T cell recruitment might contribute to the regulation of TMEM123 expression.

## Data availability statement

The original contributions presented in the study are included in the article/**Supplementary Material**. Further inquiries can be directed to the corresponding authors.

## Author contributions

EP, CC: Conceptualization, formal analysis, investigation, data curation, methodology, writing–original draft. MB, SO, CM, MC, SC, TD, MM, PG, VB and MS: Formal analysis, investigation. SE-C, CE, MCo and EDC: Formal analysis, investigation, methodology. AG: Formal analysis. GV, EC, LB: Resources. SB and SA: discussed results, provided advice and commented on the manuscript. LT: Conceptualization, resources, writing–original draft. RG: Conceptualization, formal analysis, supervision, funding acquisition, investigation, writing–original draft, project administration. All authors contributed to the article and approved the submitted version.
