# Corrigendum: TMEM123 a key player in immune surveillance of colorectal cancer

**DOI:** 10.3389/fimmu.2023.1256713

**Published:** 2023-07-28

**Authors:** Elisa Pesce, Chiara Cordiglieri, Mauro Bombaci, Serenella Eppenberger-Castori, Stefania Oliveto, Cristina Manara, Mariacristina Crosti, Caner Ercan, Mairene Coto, Andrea Gobbini, Susanna Campagnoli, Tiziano Donnarumma, Manuele Martinelli, Valeria Bevilacqua, Elisa De Camilli, Paola Gruarin, Maria L. Sarnicola, Elisa Cassinotti, Ludovica Baldari, Giuseppe Viale, Stefano Biffo, Sergio Abrignani, Luigi M. Terracciano, Renata Grifantini

**Affiliations:** ^1^Istituto Nazionale Genetica Molecolare (INGM), Padiglione Romeo ed Enrica Invernizzi, IRCCS Ospedale Maggiore Policlinico, Milan, Italy; ^2^Institute of Pathology, University Hospital Basel, Basel, Switzerland; ^3^CheckmAb Srl, Milan, Italy; ^4^Department of Pathology, European Institute of Oncology, Milan, Italy; ^5^Department of Surgery, Fondazione IRCCS Cà Granda, Ospedale Maggiore Policlinico, Milan, Italy; ^6^Department of Oncology and Hemato-oncology, Università degli Studi di Milano, Milan, Italy; ^7^Department of Biosciences, Università degli Studi di Milano, Milan, Italy; ^8^Department of Clinical Sciences and Community Health, Università degli Studi di Milano, Milan, Italy; ^9^IRCCS Humanitas Research Hospital, Rozzano, Italy; ^10^Department of Biomedical Sciences, Humanitas University, Pieve Emanuele, Italy

**Keywords:** colorectal cancer, tumor microenvironment, tumor-infiltrating lymphocytes, TMEM123, cytoskeleton organization, cell adhesion, migration

In the published article, there was an error in affiliation 9. Instead of “Anatomic Pathology Unit, Humanitas University Research Hospital, Milan, Italy”, it should be “IRCCS Humanitas Research Hospital, Rozzano, Italy”.

In the published article, there was also an error regarding the affiliations for Luigi M. Terracciano. As well as having affiliation 9, they should also have “Department of Biomedical Sciences, Humanitas University, Pieve Emanuele, Italy”.

The authors apologize for these errors and state that they do not change the scientific conclusions of the article in any way. The original article has been updated.

