# Supplementary material for: TMEM123 a key player in immune surveillance of colorectal cancer
Source: Front Immunol. 2023 Jun 23;14:1194087. doi: 10.3389/fimmu.2023.1194087 (PMC10327427; doi:10.3389/fimmu.2023.1194087)
Supplement: Supplementary file 6 [file DataSheet_1.pdf]

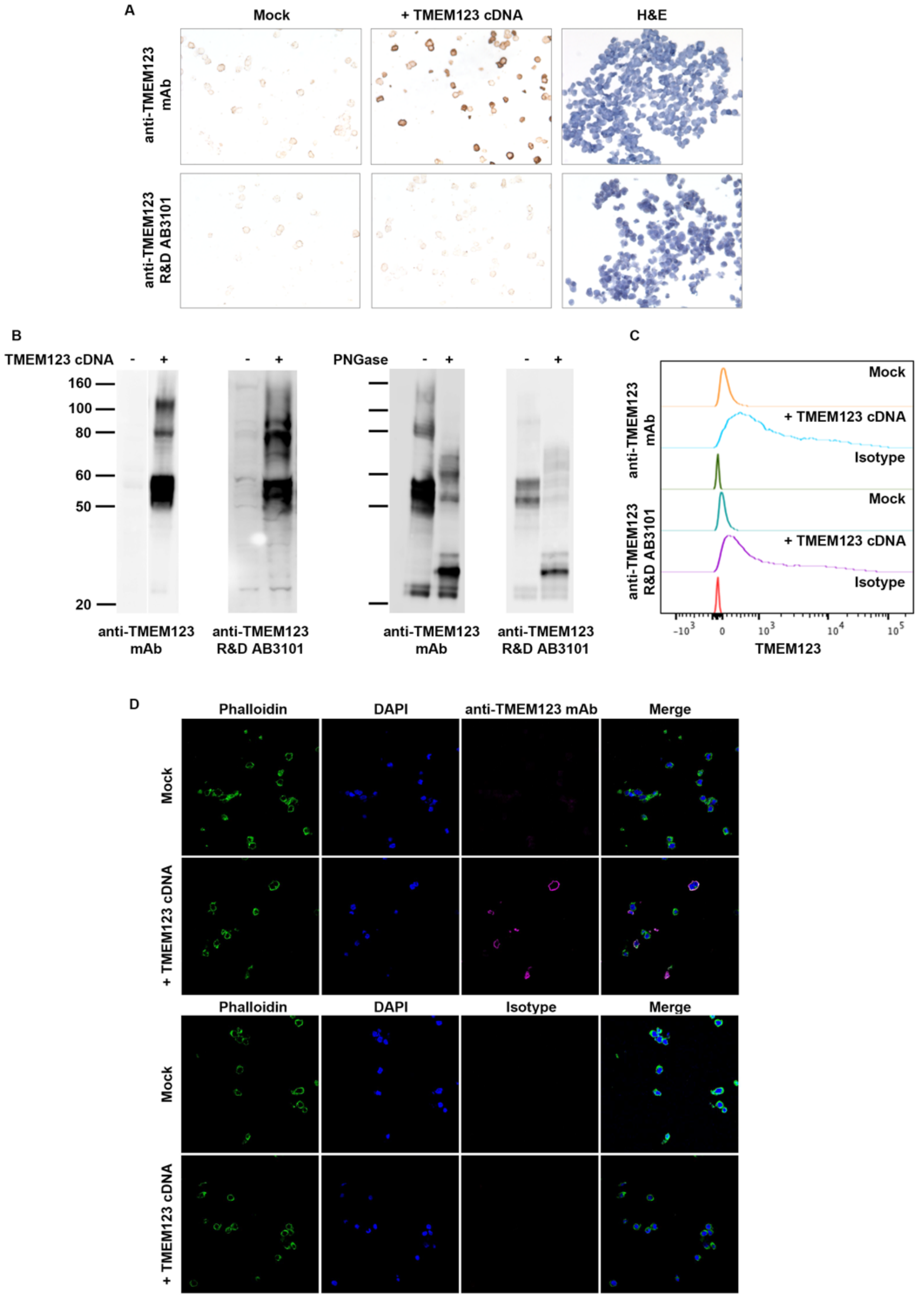

Fig. S1 The anti-TMEM123 mAb specifically recognizes the protein on the surface of HEK293T cells transfected with a plasmid encoding full length TMEM123. Hek293T cell were transiently transfected with TMEM123 cDNA or mock and incubated by ad hoc generated anti-TMEM123 mAb. **(A)** Immunocytochemical analysis of paraffin-embedded Hek293T cell pellets. **(B)** Western blot analysis. Whole-cell lysates were prepared from cells samples, separate by PAGE-SDS and used for Western blot, before (left) or after (right) enzymatic deglycosylation. The molecular mass of expressed TMEM123 ranging from 55 to 120 kDa, due to a high glycosylation state, as confirmed by PNGase treatment. (**C**) Flow cytometry analysis of mock and TMEM123 cDNA transfected Hek293T cell using anti-TMEM123 mAb or commercial antibody to detect surface expression of the protein. The results are shown as a histogram of fluorescent intensity. (**D**) Immunofluorescence staining of OCT-embedded HEK293T cell pellets transiently transfected with TMEM123 cDNA or mock using anti-TMEM123 mAb.

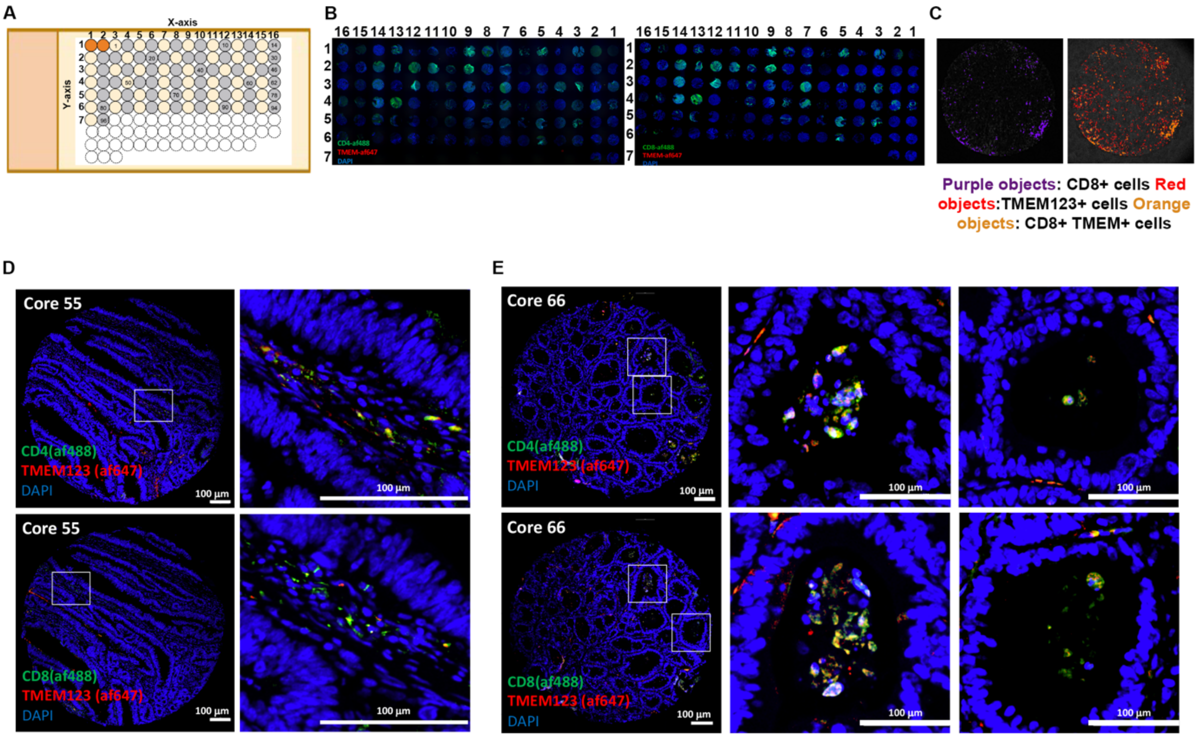

Fig. S2 Co immunofluorescence staining for TMEM123 on CD4 and CD8 T cells in tissue microarrays of colon cancer samples. (**A**) Schematic representation of ad-hoc prepared tissue micro array (TMA) of colon tissue samples for TMEM123 expression analysis, related to CD4 and CD8 T cells. (**B**) Low-magnification automated spinning disk confocal microscopy large-image of whole TMA slide labelled with anti-CD4 (left) or anti-CD8 (right) (both in green LUT), together with anti-TMEM123 (red LUT) and DAPI (blue LUT). (**C**) Representative images to highlight major binary objects segmented and classified for image analysis of all studied TMA tissue cores, by digital image analysis as to obtain quantifications of TMEM123+ cells, CD4+ or CD8+ cells, plus secondary objects containing both TMEM123 and CD4 or CD8 signals. Representative images show object segmentation example on one tissue core co-labelled for TMEM123 and CD8, where all segmented classified objects for CD8 signal are shown in purple, objects for TMEM123 signal in red and objects classified for both TMEM123 and CD8 signals are displayed in orange. (**D-E**) Representative images acquired via automated large-images (each image is a mosaic of 25 FOVs at 20x, acquired at best focal plan) and at higher magnification (40x) of details of interest, via laser scanning confocal microscopy from n=2 representative analysed cores of colon cancer tissues (core 55, D and core 66, E), immunolabelled for CD4 (af488; upper row) or CD8 (af488; lower row), both displayed in green LUT, together with anti-TMEM123 labelling (af647; red LUT) and nuclear DAPI counter-staining. Yellowish coloring corresponds to co-presence of both TMEM123 and CD labelling. Magnification bars: 100 µm for all images.

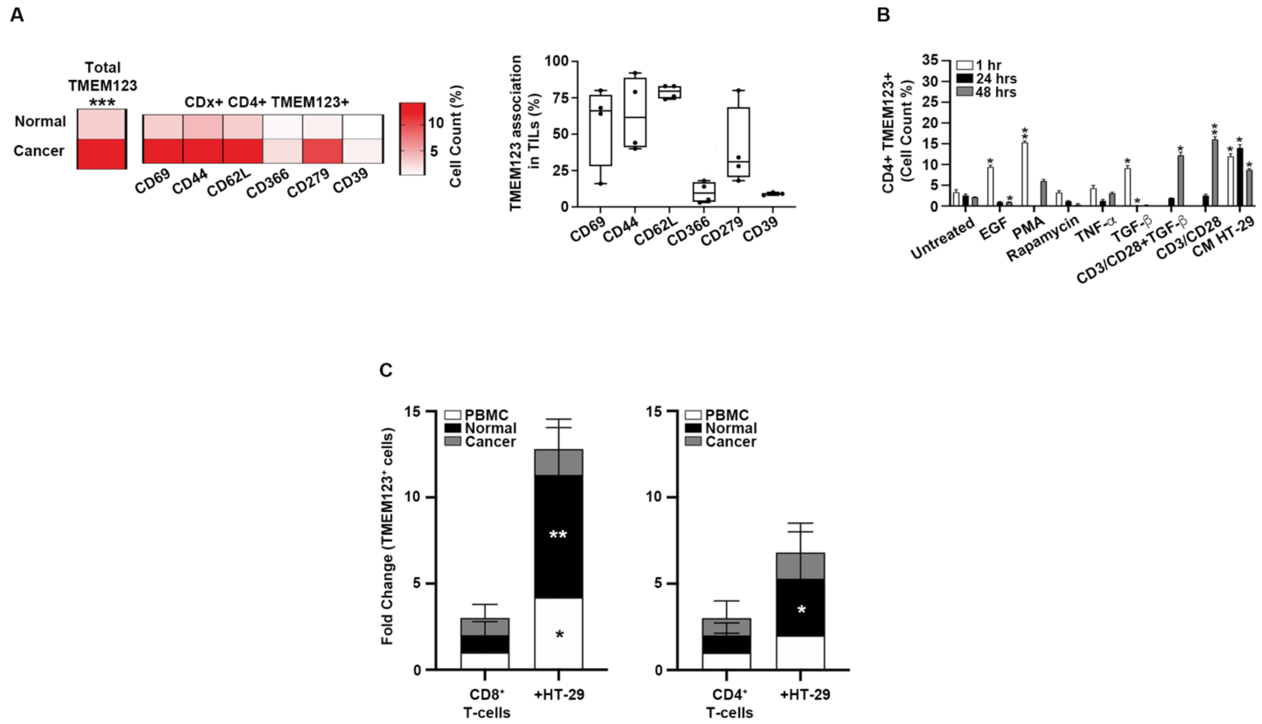

Fig. S3 TMEM123 is co-expressed with activation markers in CD4 T cells and is induced by microenvironmental stimuli. (**A**) FACS analysis of TMEM123 in tumour infiltrating CD4+ T lymphocytes for surface expression of indicated markers. Heat-maps show the mean of 4 independent experiments. The graph shows the percentage of association of TMEM123 with each marker, in CD4+ T cells. (**B**) PBMC from healthy donors were incubated with EGF, PMA, rapamycin, TNFα, TGFβ, CD3/CD28 or with the conditioned media (CM) of cancer cells (HT-29) for 1h (white), 24h (black) and 48h (grey) and the presence of TMEM123 on the surface of CD4+ T cells was followed by FACS analysis normalizing on the untreated samples. (**C**) CD4+ and CD8+T cells were isolated from CRC (grey), normal colon (black) and PBMC (white) and culture alone or co-cultured with HT-29 cells at ratio 10:1 for 48 hours. Lymphocytes were stained with anti-TMEM123, anti-CD8 and anti-CD4 mAbs (left and right panels, respectively) for FACS analysis.

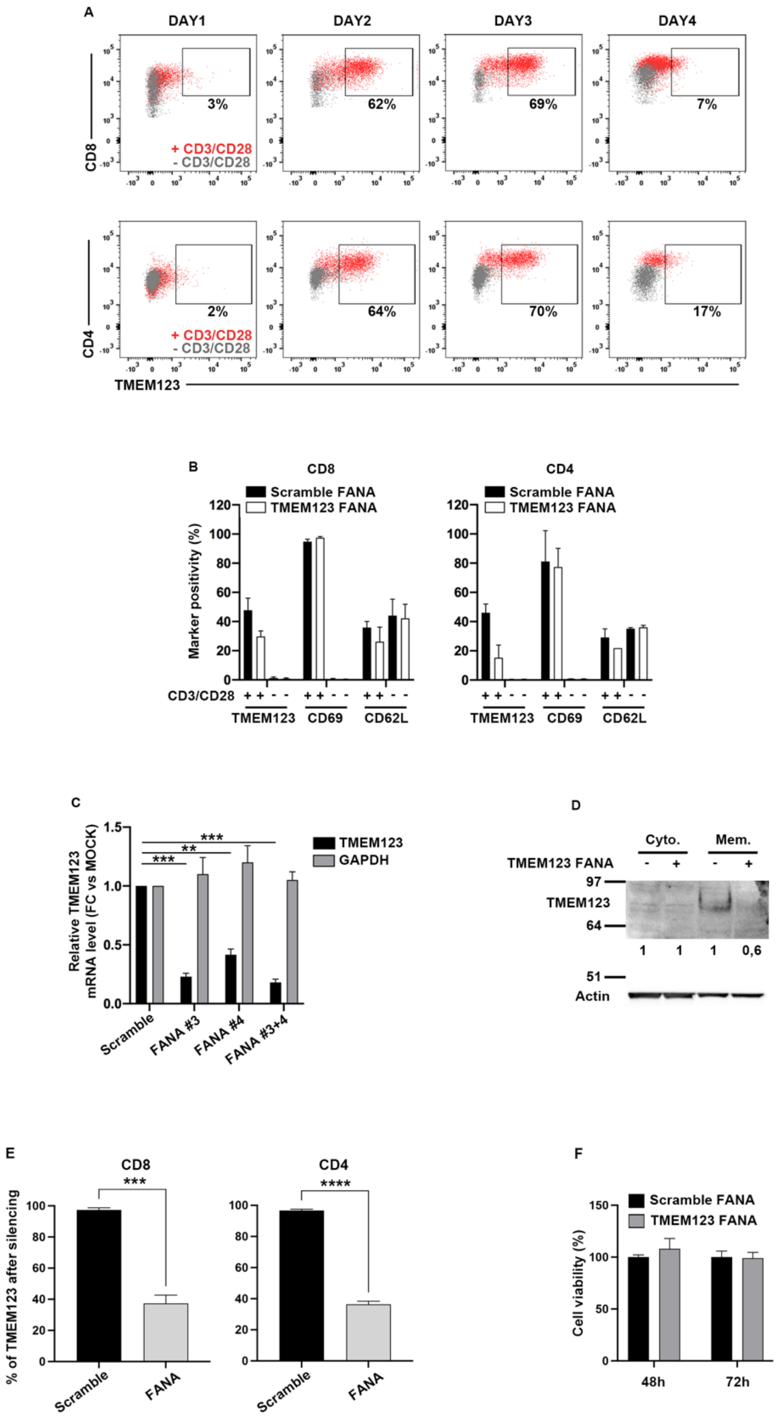

Fig. S4 TMEM123 is upregulated after TCR stimulation. (A) CD8 (upper panel) and CD4 (lower panel) were isolated from PBMC of HD and activated or not with CD3/CD28 Dynabeads for up to 4 days. At the indicated time points, T cells were harvested and stained with anti-CD4, anti-CD8 and anti TMEM123 mAbs for FACS analysis. (**B)** CD8 (left) and CD4 (right) T cells were isolated from PBMC of HD and activated or not with CD3/CD28 Dynabeads and treated with TMEM123 FANA-aso oligonucleotides (white) or scramble FANA (black). After 2 days T cells were stained with TMEM123, CD69 or CD62L mAbs and analysed by FACS. (**C-F**) TMEM123 silencing in Jurkat and T cells with TMEM123-specific FANA-aso antisense oligonucleotides. Jurkat cells or T lymphocytes were incubated for 2 days in the presence of two different TMEM123 FANA-aso oligonucleotides (as single or in combination) or with scramble FANA-aso at 10 µM concentrations. The loss of TMEM123 expression in Jurkat cells was assessed by qRT-PCR (**C**) and western blot of the subcellular fractionation (**D**) as compared to mock-treated samples. (**E**) Loss of TMEM123 expression in CD8 or CD4 T cells, assessed relatively to the TMEM123-positive cells. The reduction of TMEM123 expression (using the combination FANA #3+#4) was assessed by FACS analysis. (**F**) Viability of CD8 T cells treated with TMEM123-FANA or scramble was monitored over time with the PrestoBlue reagent. Values expressed in % as mean ± SD. Statistical significance is denoted by asterisks (*= <0.05; **= 0.01; ***< 0.001; ****= <0.0001).

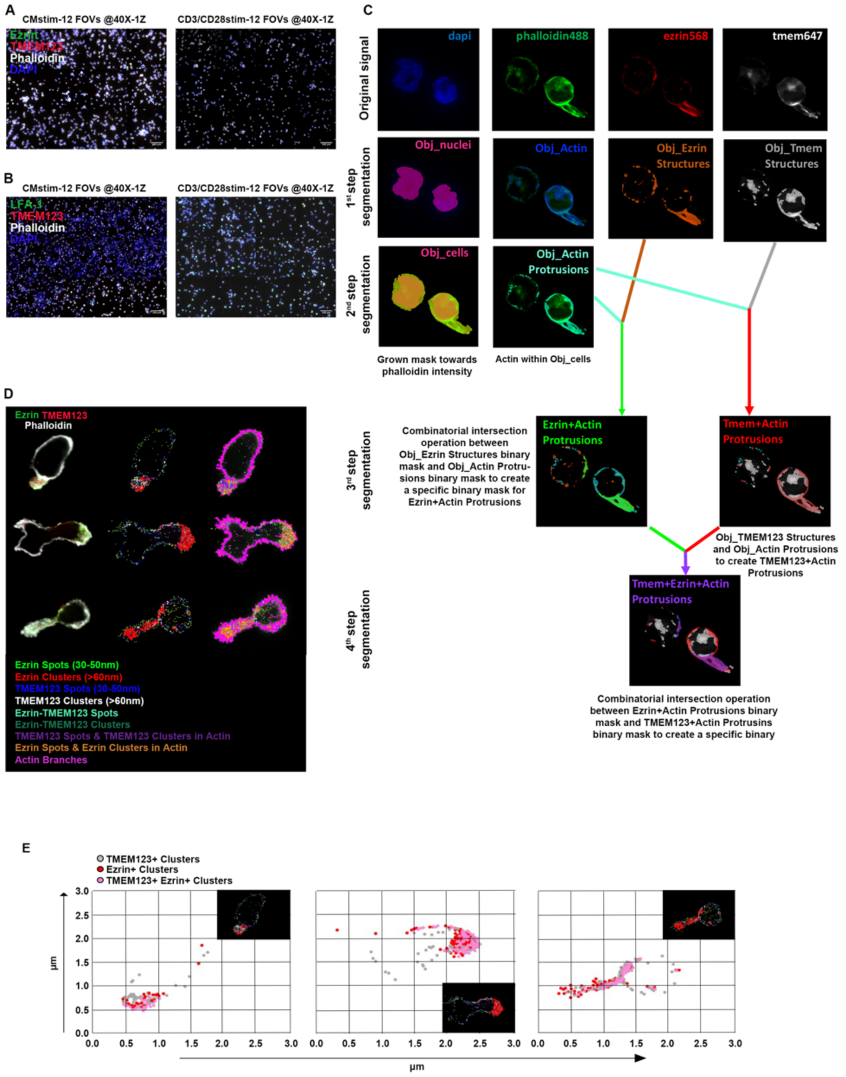

Fig. S5 TMEM123 co-expressed with Ezrin and LFA-1: pipelines of analysis. Image analysis of Jurkat cells stimulated with CM or CD3/CD28 for in vitro protrusion formation over poly-L-lysine coated glass-bottom chamber-slides. (**A-B)** Acquisitions were performed on multiple Z-stacks at 40x and 100x magnification with spinning disk confocal microscopy. Best focal plan for actin phalloidin staining was chosen on all acquired FOVs (n=15 fovs/sample/ condition) and the best 12 FOVs were chosen and mounted as multiposition-montage for representative image of high cell numbers/sample. (**C)** Schematic explanation of cascade sequential segmentation strategy to evaluate and quantify the cellular structures positive for TMEM123 signal in combination with the signal of the uropodal marker Ezrin. The analysis pipeline was conducted using the General Analysis module with Nis-Elements software (Nikon-Lim), using machine learning and artificial intelligence tools for best thresholding and segmentation watershedding. Pipelines of analysis were always set-up using positive control images, tested on negative control images, first run-in supervised manner on selected fields of view and then applied in unsupervised-automatic fashion in batch, as to lead to an unbiased image quantification. Automated segmentation analysis was performed on all images accounting for a total of n=432 cells from n=15 FOVs acquired on n=4 independent biological replicates. Similar sequential segmentation pipeline of analysis was also conducted for LFA-1 marker. (**D)** n=52 cells, acquired by 775nm 2-color STED microscopy with 25nm pixel size sampling for correct 50nm resolution, were analysed for single protein spots and molecule clustering. Binary layers over images as evaluated by single molecule analysis, to quantify single Ezrin Spots (range 30-50nm diameter; green objects), Ezrin Clusters (range >60nm diameter, red objects), single TMEM123 Spots (range 30-50nm diameter; blue objects), TMEM123 Clusters (range >60nm diameter, white objects), Ezrin-TMEM123 Spots (light-blue objects), Ezrin-TMEM123 Clusters (dark-green objects), TMEM123 Spots and TMEM123 Clusters localized within Actin Branches (purple objects), Ezrin Spots and Ezrin Clusters localized within Actin Branches (orange objects), Actin Branches (magenta objects). (**E)** Spatial distribution of clusters over the cellular area. Three representative cells are shown to depict the spatial proximity of clusters of molecules, which tend to preferentially localize in one particular cell edge, which show distinct morphology, depending on cell spatial spreading and cytoskeleton organization, including polarized edges of cell (leftt and central panel) or cellular protrusions (right panel).

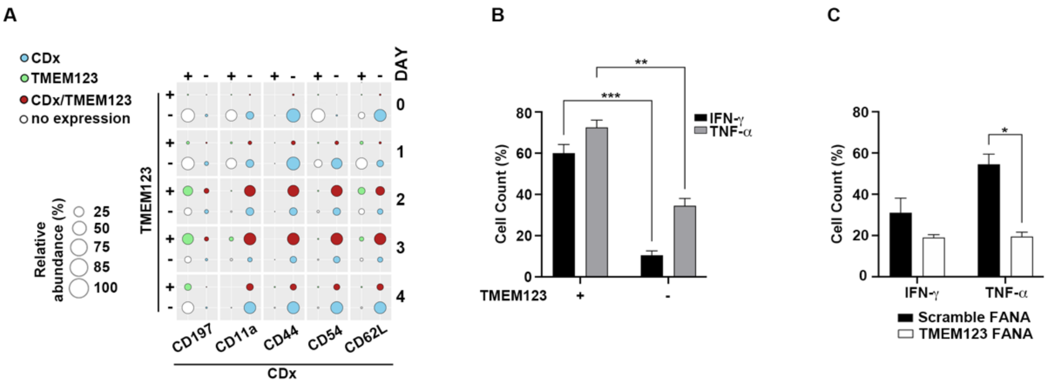

Fig. S6 TMEM123 is co-expressed with known T lymphocytes markers highly enriched in T cell adhesion and migration. (A) Dot plot represents FACS analysis on CD4+ T cells for TMEM123 expression as standalone or in co-expression with tested markers. Data represent the average of at least 3 experiments. (**B**) TMEM123 positive CD4 T cells express high level of effector cytokines. CD3/CD28 activated CD4 T cells from PBMC of HD were treated with PMA-ionomycin-brefeldin-A and stained for indicated cytokines. Graph reports the percentage of cytokine produced from TMEM positive or TMEM negative T cells as determined by FACS analysis. (**C**) TMEM123 silencing impairs production of effector cytokines. CD3/CD28 activated CD4 T cells were further treated with TMEM123-FANA and cytokine expression was assessed by FACS. Statistical significance is denoted by asterisks (p-value *= <0.05; **= <0.01).

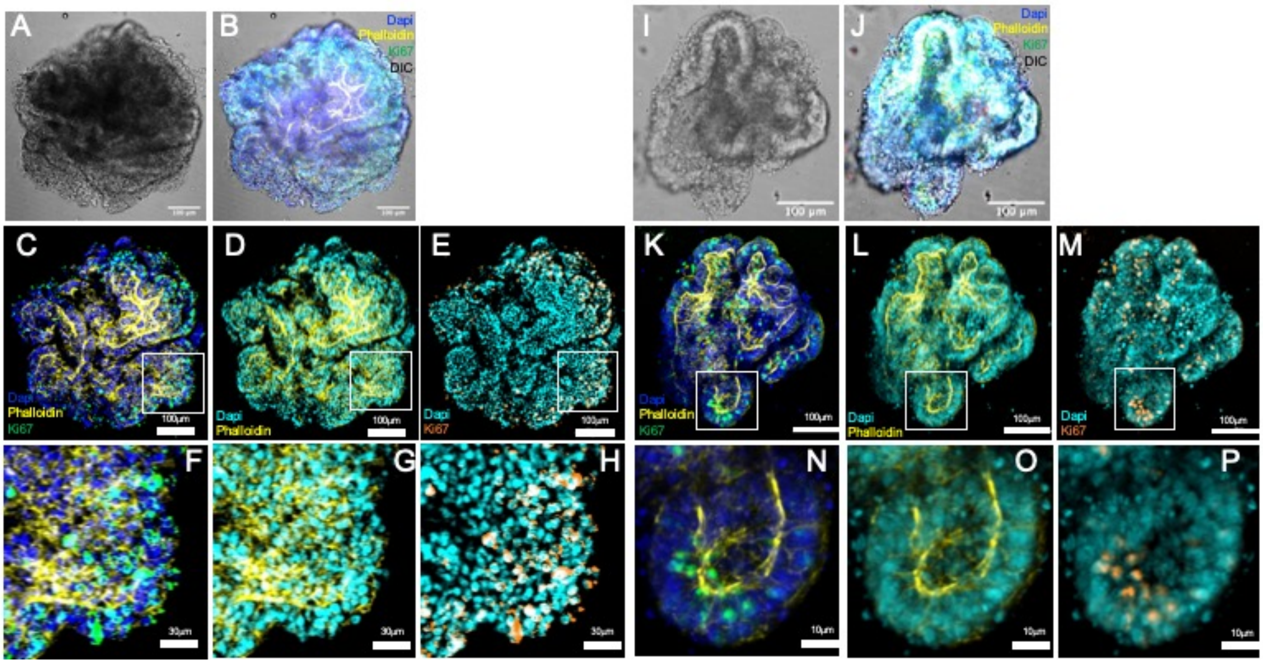

Fig. S7 Representative tumoroids without T cell co-culture used as internal control. Images show two different tumoroids undergoing same culturing timing and conditions, but without co-presence of T lymphocytes (A to H, left panel tumoroid and I to P, right panel tumoroid), following whole-mount immune-labelling. Panels show organoids in differential interference contrasted transmitted light bright-field imaging, using a 10x magnification without (A, I) or with (B, J) superimposed fluorescence channels merging in spinning disk confocal microscopy acquisition at specific best focal plans, plus detailed high resolution fluorescence images acquired at two different magnifications with laser-scanning confocal microscope (C-H and K-P). Organoids were labelled for cytoskeleton (phalloidin, yellow), for proliferating cells (anti-Ki-67, green in B,C,F and J,K,N and orange in E,H and M,N) and nuclei were counterstained with Dapi (blue in B,C,F and J,K,N or cyan in D,E,G,H and L,M,O,P). Scale bars in images: 100, 30 and 10 µm.

Table S1. Clinical and pathological characteristics of the colon cancer patients' cohorts and corresponding tissue on TMAs

Characteristics	CRC TMA 1	TMA Biomax	CRC TMA 2
**Number of patients**	46	40	43
**Number of cores**	59	50 x 2	96
**Patients' Age (y) mean (min-max)**	71 (38 – 91)	59 (26-82)	66 (42 – 87)
**Patients' Gender: Female / Male**	19 / 27	20 / 20	18 / 25
**pT 1**	1	0	1
**pT 2**	7	2	4
**pT 3**	31	36	30
**pT 4**	7	2	8
**pN 0**	25	12	16
**pN 1**	13	27	24
**pN 2**	8	1	3
**pM 1**	5	1	15
**Grade 1**	1	2	0
**Grade 2**	38	22	37
**Grade 3**	7	10	6
**Primary CRC**	46	40	43
**Non malignant adjacent tissue**	13	10	0
**Metastatic or relapse tissue***	0	0	53
**Overall Survival (Months) Mean (min-max)**	52 (1-199)	NA	66 (8-235)

***The metastatic or relapse tissue on CRC TMA 2 were all paired to the primary CRC with the following details: 8 metastases syncron with primary CRC; the remaining were longitudinal samples: 6 recurrent CRC and 39 metastases.**

CRC TMA 1: built at the biobank of pathology University Hospital Basel, Ethical approval EKBB 361/12 by means of a TMA-Grand Master Semiautomatic arrayer of 3D-Histeck, diameter of each core: 1 mm

TMA BIOMAX: commercial TMA, reference: Biomax, inc Cat N° CO1004, link: https://www.biomax.us/tissue-arrays/Colon/CO1004

CRC TMA 2: built within the same project of the CRC TMA 1 at the biobank of pathology University Hospital Basel

Table S2. Clinical characteristics of cancerous tissues and adjacent normal tissues samples obtained from surgical resections of oncologic patients used for *ex vivo* analysis

Tissue ID	Matrix Sub-Type	Matrix Type	Division	Snomed T	Snomed M	Grade	pT	pN	M	Disease State	Snomed T Code	Snomed M Code	Age	Sex
18-C-00037-01	P	Colon	A-P-S	Colon, NOS	Adeno., NOS	3	3(m)	2a		Primary	T-59300	M-81403	62	F
18-C-00038-01	NP	Colon	A-P-S	Colon, NOS	Adeno., NOS	3	3(m)	2a		Primary	T-59300	M-81403	62	F
18-C-00041-01	P	Colon	A-P-S	Stomach, NOS	Adeno., NOS	2	3	2		Primary	T-57000	M-81403	79	F
18-C-00042-01	NP	Colon	A-P-S	Stomach, NOS	Adeno., NOS	2	3	2		Primary	T-57000	M-81403	79	F
18-C-00045-01	P	Colon	A-P-S	Colon, NOS	Adeno., NOS	3	3	2b		Primary	T-59300	M-81403	76	M
18-C-00046-01	NP	Colon	A-P-S	Colon, NOS	Adeno., NOS	3	3	2b		Primary	T-59300	M-81403	76	M
19-C-00026-01	P	Colon	A-P-S	Colon, NOS	Adeno., NOS	3	4a	1b		Primary	T-59300	M-81403	84	F
19-C-00026-03	P	Colon	A-P-S	Colon, NOS	Adeno., NOS	3	4a	1b		Primary	T-59300	M-81403	84	F
19-C-00027-01	NP	Colon	A-P-S	Colon, NOS	Adeno., NOS	3	4a	1b		Primary	T-59300	M-81403	84	F
19-C-00035-01	P	Colon	A-P-S	Colon, NOS	Adeno., NOS	2	3	0		Primary	T-59300	M-81403	86	M
19-C-00036-01	NP	Colon	A-P-S	Colon, NOS	Adeno., NOS	2	3	0		Primary	T-59300	M-81403	86	M
19-C-00041-02	P	Colon	A-P-S	Colon, NOS	Adeno., NOS	3	3	0		Primary	T-59300	M-81403	79	F
19-C-00041-03	P	Colon	A-P-S	Colon, NOS	Adeno., NOS	3	3	0		Primary	T-59300	M-81403	79	F
19-C-00042-03	NP	Colon	A-P-S	Colon, NOS	Adeno., NOS	3	3	0		Primary	T-59300	M-81403	79	F
19-C-00104-01	P	Colon	A-P-S	Colon, NOS	Adeno., NOS	2	3	0		Primary	T-59300	M-81403	45	F

19-C-00105-01	NP	Colon	A-P-S	Colon, NOS	Adeno., NOS	2	3	0		Primary	T-59300	M-81403	45	F
19-C-00108-01	P	Colon	A-P-S	Colon, NOS	Adeno., NOS	3	3	0		Primary	T-59300	M-81403	82	F
19-C-00109-01	NP	Colon	A-P-S	Colon, NOS	Adeno., NOS	3	3	0		Primary	T-59300	M-81403	82	F
19-C-00110-01	P	Colon	A-P-S	Colon, NOS	Adeno., NOS	2	2	0		Primary	T-59300	M-81403	84	M
19-C-00111-01	NP	Colon	A-P-S	Colon, NOS	Adeno., NOS	2	2	0		Primary	T-59300	M-81403	84	M
19-C-00112-01	P	Colon	A-P-S	Colon, NOS	Adeno., NOS	2	1	0		Primary	T-59300	M-81403	81	F
19-C-00113-01	NP	Colon	A-P-S	Colon, NOS	Adeno., NOS	2	1	0		Primary	T-59300	M-81403	81	F
20-C-00016-02	P	Colon	A-P-S	Colon, NOS	Adeno., NOS	3	3	2b		Primary			68	F
20-C-00018-02	NP	Colon	A-P-S	Colon, NOS	Adeno., NOS	3	3	2b		Primary			68	F
20-C-00001-02	P	Colon	A-P-S	Rectum, NOS	Adeno., NOS	2	3	0		Primary			68	F
20-C-00003-02	NP	Colon	A-P-S	Rectum, NOS	Adeno., NOS	2	3	0		Primary			68	F
19-C-00102-01	P	Colon	A-P-S	Rectum, NOS	Adeno., NOS	2	1	0		Primary			80	M
19-C-00101-01	NP	Colon	A-P-S	Rectum, NOS	Adeno., NOS	2	1	0		Primary			80	M
